# Early warning of complex climate risk with integrated artificial intelligence

**DOI:** 10.1038/s41467-025-57640-w

**Published:** 2025-03-15

**Authors:** Markus Reichstein, Vitus Benson, Jan Blunk, Gustau Camps-Valls, Felix Creutzig, Carina J. Fearnley, Boran Han, Kai Kornhuber, Nasim Rahaman, Bernhard Schölkopf, José María Tárraga, Ricardo Vinuesa, Karen Dall, Joachim Denzler, Dorothea Frank, Giulia Martini, Naomi Nganga, Danielle C. Maddix, Kommy Weldemariam

**Affiliations:** 1 Amazon Web Services, Seattle and Santa Clara, WA and CA USA; 2 ELLIS Unit Jena, Jena, Germany; 3 Max-Planck-Institute for Biogeochemistry, Jena, Germany; 4 ETH Zurich, Zurich, Switzerland; 5 University of Jena, Jena, Germany; 6 University of Valencia, Valencia, Spain; 7 Potsdam Institute for Climate Impact Research, Potsdam and Berlin, Germany; 8 University of Sussex, Sussex, UK; 9 University College London, London, UK; 10 Lamont-Doherty Earth Observatory, Columbia University, New York, NY USA; 11 International Institute for Applied Systems Analysis (IIASA), Laxenburg, Austria; 12 Max-Planck-Institute for Intelligent Systems, Tübingen, Germany; 13 ELLIS Institute Tübingen, Tübingen, Germany; 14 KTH Royal Institute of Technology, Stockholm, Sweden; 15 German Red Cross, Berlin, Germany; 16 World Food Program, Rome, Italy; 17 Kenya Red Cross, Nairobi, Kenya

**Keywords:** Environmental impact, Natural hazards

## Abstract

As climate change accelerates, human societies face growing exposure to disasters and stress, highlighting the urgent need for effective early warning systems (EWS). These systems monitor, assess, and communicate risks to support resilience and sustainable development, but challenges remain in hazard forecasting, risk communication, and decision-making. This perspective explores the transformative potential of integrated Artificial Intelligence (AI) modeling. We highlight the role of AI in developing multi-hazard EWSs that integrate Meteorological and Geospatial foundation models (FMs) for impact prediction. A user-centric approach with intuitive interfaces and community feedback is emphasized to improve crisis management. To address climate risk complexity, we advocate for causal AI models to avoid spurious predictions and stress the need for responsible AI practices. We highlight the FATES (Fairness, Accountability, Transparency, Ethics, and Sustainability) principles as essential for equitable and trustworthy AI-based Early Warning Systems for all. We further advocate for decadal EWSs, leveraging climate ensembles and generative methods to enable long-term, spatially resolved forecasts for proactive climate adaptation.

## Abstract (web summary)

In the era of climate change, human societies face growing exposure to disasters and complex climate risks. This perspective explores the transformative potential of integrated Artificial Intelligence in developing multi-hazard Early Warning Systems for all.

## Introduction

Early-warning systems (EWS) are an essential component of risk-reduction strategies for climate and environmental hazards and thus should be a central element of resilient sustainable-development strategies^1^. The United Nations (UN) and the World Meteorological Organization (WMO) recognize the importance of these EWS and have installed efforts to develop them via the Early-Warnings-for-All Initiative launched in 2022, also related to Target G of the UN Sendai Framework 2015–2030^2^. There are numerous past cases proving the value of EWSs for saving lives and livelihoods^3–5^. One key example is the investment in research and implementation in tsunami warnings following the Indian Ocean tsunami in 2004^6^. Focused collaboration has resulted in more robust, international, and technologically advanced warnings that have saved many lives since 2004, including during the 2011 Tōhoku tsunami^7^. However, it is essential to recognize that complex risks from surging climate and weather extremes involving multiple hazards as either concurrent or cascading events pose significant additional challenges to developing effective EWS in particular when considering the non-stationary conditions of many climate impact drivers due to anthropogenic climate change^8^: Projected increases in severity and frequency under unmitigated greenhouse gas emissions on top of changing exposure and vulnerability will make these efforts even more important for future climate risk adaptation^9^, because relying on past norms and guidelines will prove inappropriate under non-stationary risks.

The United Nations^10^ define EWS as *‘An integrated system of hazard monitoring, forecasting and prediction, disaster risk assessment, communication and preparedness activities, systems and processes that enables individuals, communities, governments, businesses and others to take timely action to reduce disaster risks in advance of hazardous events’*. Yet, current EWS tend to emphasize the hazard prediction (e.g., weather) compared to impact prediction and communication (a recent example is the Ahr Flood in Germany (cf. SI Table 1)), where weather forecast and weather warning from the weather services was timely and correct, but the impact not anticipated^11^ and adequate preventive measures not taken. Lately, there has been more focus towards impact-based forecasts and warnings (IBFW)^12^. However, early studies suggest that these make little difference to outcomes as they still only provide the impact information rather than what actions to take in response^13^. Moreover, it has been proposed to design IBFWs for individual members of the public, which allows for a more fine-grained treatment of vulnerability and coping capacity, thus increasing the strength of future warnings^14^.

The accuracy and effectiveness of EWSs depend not just on the quality of data gathered from sensors, process understanding, and the ability to predict hazards accurately and assess their potential impact, but also the speed and effectiveness of communication, and the ability to make timely and effective decisions, e.g., implemented as Anticipatory Action in the humanitarian domain^15^. All of this requires preparedness to enable the EWS to be sustainable, effective, and enable the end users to take early actions to enhance their safety and reduce economic and social losses. An important aspect for EWS is the relevant time-scale. Early warnings typically vary from seconds, to tens of thousands of years, but for climatic hazards time scales are generally on the hourly to weekly time-scale for more rapid onset hazards (e.g., storms), and have longer time scales for slow-onset hazards (e.g., drought, desertification). Longer time scales beyond a year are not considered on classical EWS but are very relevant for conscious spatial and infrastructure planning and societal preparedness, especially in the context of climate change^16^. The diversity of relevant aspects for early warning results in tremendous complexity and challenges in implementing effective EWS, while some require a large collective effort to make progress, for others, AI can offer the necessary leap forward.

## Main challenges with early-warning systems

**Fig. 1:**
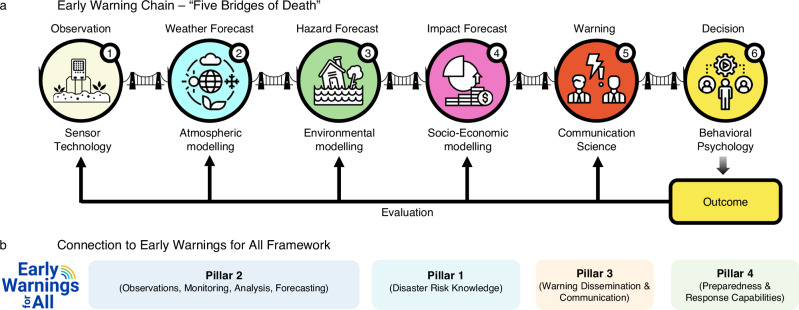
Depiction of the early-warning chain from observation to decision and its relation to the Early Warnings for All Framework. **a** “All five bridges of death” (pers. comm. Brian Golding, HiWeather) have to be crossed for an effective early warning. Figure modified after ref. ^17^. **b** Link to the four pillars of the UN Early Warnings for All Framework.

Weather-related EWSs operate along a warning chain involving observations, forecasts (weather, hazard, impact), communication and decision-making^17^, and should be continuously evaluated (Fig. 1). As in the case of a chain, the overall efficacy largely depends on the weakest link, which would then undermine skill and technical advancement in others.

**Fig. 2:**
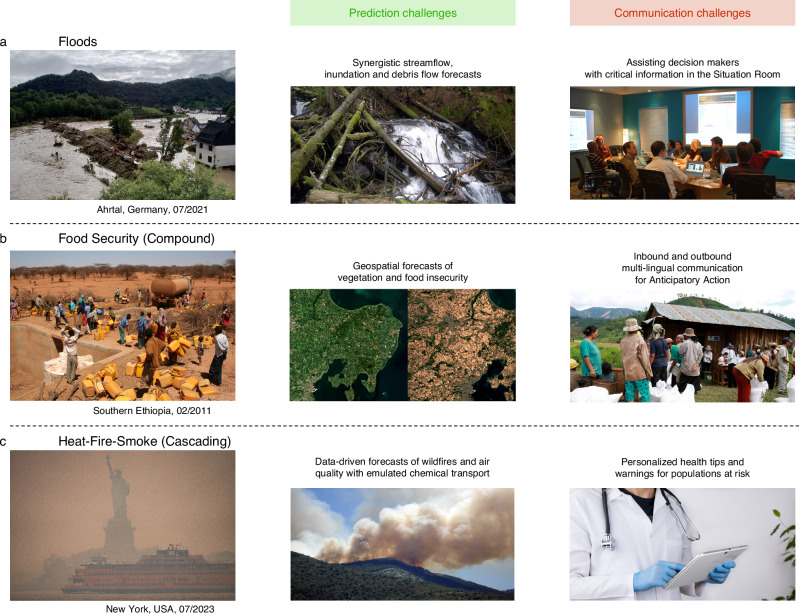
Three cases of past disasters and where machine learning could have helped in either prediction, communication, or both. **a** The European Ahrtal flood, **b** drought-related food insecurity in Eastern Africa, **c** heat-fire-smoke hazard in North America. For a detailed description, see Table S1. This figure uses images licensed under CC BY 2.0, detailed image credits are listed in the “Acknowledgements” section.

Hydrometeorological hazard forecasts to a large extent rely on numerical weather prediction (NWP), which has improved tremendously over the past decades^18^. However, challenges remain: For example, with fast-onset disasters such as storms and floods, accurate forecasting of precipitation is necessary, a fact that involves resolving convection, which is computationally slow in NWP models. Hence, for such disasters, lead times can be fairly short, sometimes too short for effective action^19^. For instance, in late 2021, the tropical cyclone Rai struck the Philippines. It had undergone an unforeseen very rapid intensification in the hours before landfall, a lead time that makes it difficult to still start activities that are anticipatory in nature^20^. On the other hand, early warning for slow-onset disasters, e.g., droughts, builds upon sub-seasonal to seasonal forecasting^21^. At these time scales, predictability is driven by boundary conditions such as the sea-surface temperatures and the land-surface soil moisture and with the chaotic nature of the atmosphere, seasonal forecasts suffer from a lot of uncertainty (cf. Fig. 2, Table S1), although for large-scale extremes like the recent long lasting Horn of Africa drought some predictive skill has been achieved^22^. Yet for more localized extremes, given uncertain boundary conditions and the chaotic nature of the weather system, there is seldom enough forecast certainty on spatio-temporal extents to enable effective early action more than a few weeks in advance.

Crucially, weather or hazard forecasts are not sufficient, because the same weather event can result in vastly different impacts. For instance, this was evidenced in Germany 2021 (Fig. 2, Table S1), where a few weeks before the devastating Central European floods there was a similar meteorological event in North Eastern Germany with almost no impact^23^. The reason is a completely different landscape, which is less hilly and has more sandy soils, allowing for faster infiltration of rain. Yet predictions of impacts are challenging because they result from the interaction of the weather system with ecosystems/landscapes and societal systems^24^. This needs to consider sub km-scale, often m-scale, local context, and variables that are outside the physical climate system. In addition, even hydrodynamic models partially failed in 2021 because the complexities such as debris flow and geohydromorphological dynamics have not been considered. Moreover, societal impact forecasts need to build upon maps of exposure and vulnerability^4^. Such maps are often coarse in resolution due to a general sparsity of gridded socioeconomic data, although down-scaling attempts have yielded promising results^25^. Combining them with the physical variables obtained from the hazard forecast is not trivial, especially since dynamic aspects of vulnerabilities are not often considered^26^.

Another major challenge is concurrent, compounding, and cascading events, hampered by the lack of connections across the various thematic, institutional, and regional silos^27^, but also because forecasts across systems are harder, because of complexity, and if interdisciplinary model integration is lacking. This is particularly critical, as amplifying cross-border effects such as impacts on supply chains, water management and disaster response capacities are important. Even more broadly, teleconnections, e.g., due to trade, river systems and atmospheric transport, are barely integrated into EWS, as their inclusion requires advanced data sharing, real-time communication, and predictive models that can account for these long-distance relations, information sources and impacts^28^.

Furthermore, an ideal EWS strives to harness the full spectrum of available observations, yet present systems face notable limitations in achieving this. For instance, feeding satellite information to a physical model often requires an observational operator, as what is measured is only a proxy of what is modeled. This becomes increasingly challenging in regimes with low signal-to-noise ratio, uneven and sparse data sampling, scarcity of measurements, and wide diversity of quality, quantity and granularity of data. Hence, existing EWS rarely leverage all available data. For instance, EWS for floods and storms do not assimilate all locally available radar, gauge, and satellite information, but instead focus on a few data modalities and resolutions^29^. Furthermore, potentially informative sources of data for food security EWS, e.g., from social media or economic factors, are not typically exploited in their entirety^30^.

Communication of warnings, especially to the affected population, i.e. the last mile^12^, is another critical aspect^31^ (Fig. 2, Table S1). Numerous cases over history demonstrate that even if the EWS forecasting part produces actionable forecasts, communication failed^32^. Most recently, the Mediterranean storm Daniel led to severe rainfall and flooding in Libya with over 4300 people dead and many more displaced^33^. While a lack of communication was certainly not the only reason for this devastating outcome, it surely contributed, given even TV weather reports predicted the landfall at least four days ahead^34^. Global initiatives such as the Common Alerting Protocol have been useful to standardize warning data enabling media outlets and cell phone broadcasters to issue warnings^35^. Still, affected communities can have very different needs on EWS information, which can be best achieved by involving them already in the creation of the EWS^36^, yet doing that on a global scale is hard. Here, an additional opportunity arises, often local traditional or indigenous knowledge is overseen in EWS, but can be a useful source^37^, for instance, when it comes to the challenge of inclusiveness. Warnings should be inclusive, not just for direct ethical reasons, but also because inclusive EWSs help to save more lives, preserve livelihoods and prevent greater economic losses, and impact longer-term equitable growth and prosperity. Designing inclusive EWSs requires considering the peculiarities of diverse communities and their needs, ideally through their involvement from the beginning^38^. This is a challenge for current EWS, as adapting them to local conditions is costly, continuously iterating them through adaptive learning often restricted by rigid constraints, and in contrast, a one-size-fits-all model is cheap to implement and maintain, but not optimal for the local communities.

Last but not least, an ideal EWS should also take the expected impact of decisions based on the warnings into account, i.e., the response itself as a risk factor^39^. This can lead to a highly non-trivial decision-making feedback loop. In other words, based on the decisions that are taken upon a warning, vulnerabilities and impacts may change, which therefore would change the warning. For example, a hurricane impact forecast would be most useful if it considered not only the number of people affected by flooding and wind damage if no action were taken, but also how these numbers would change if evacuation measures were implemented. This would account for the effects of actions such as road congestion, the availability of shelters, and the potential risks posed by moving vulnerable populations. This requires the modeling of sociology and psychology, which, especially in combination with physical modeling, is challenging. Furthermore, after a disaster or an avoided disaster, the effect of interventions needs to be understood. Essentially, this requires constructing models that can operate with counterfactuals to compare “what would have happened” with “what actually happened”. For example, the food security impact of droughts is often dampened by the markets through food imports. Given this mediating effect, it is challenging to estimate what the impact of an EWS and derived anticipatory action is. Here, Earth Observation (EO) has been identified as a critical tool for Monitoring, Evaluation, Accountability, and Learning (MEAL) in the context of anticipatory action^40^. EO offers unique capabilities, such as high-resolution satellite imagery and near-real-time data, to assess changes in environmental and socioeconomic conditions both before and after an intervention. For example, satellite-derived vegetation indices can provide insight into crop health or pasture recovery, helping to infer the impact of drought-related early actions on food security. However, while EO shows great promise for strengthening the evidence base via rapid, low-cost assessments, further efforts are needed to turn these concepts into real and operational practices.

In summary, current EWSs face challenges including limitations in forecasting accuracy for fast and slow-onset disasters, difficulties in predicting impacts due to local environmental and societal variables, underutilization of diverse data sources, and challenges in effectively communicating warnings to varied communities. In addition, the complex task of incorporating societal and psychological aspects of potential warnings into the decision-making process is critical. In all of these challenges, developments in AI promise to advance the field.

## Artificial intelligence for improved early warning

Extracting knowledge from data is now increasingly achieved with techniques known as machine learning (ML) within the broader field of artificial intelligence (AI)^41^. These provide algorithms to assess the data instead of carefully chosen models. Most common is supervised machine learning, where the algorithm produces an input-output map and is trained on many labeled input-output pairs. For example, modern smartphone cameras detect faces to set the correct focus, the ML model behind it has been trained on a large dataset of pictures (inputs) and bounding boxes of the faces (output). Prevalent algorithms include variants of deep neural networks (e.g., Transformers, LSTMs or CNNs) or of decision trees (e.g., random forest or gradient boosting trees). Beyond supervised learning, there are many other ML approaches, e.g., unsupervised learning, reinforcement learning or causal inference (for a practical overview see ref. ^42^). For EWS, supervised ML will often bring the largest benefits, allowing for forecasting, mapping or also generative AI, that is e.g., generating text or image data.

### Pushing forecast accuracy

Forecasting models are available for essentially all climate hazards. Now, ML is increasingly leveraged for this purpose. As long as sufficient data is available and an input-output mapping can be defined, a ML model can be trained. In many cases, these ML models display a higher accuracy, as they can exploit statistical correlations beyond process-based theory.

In weather forecasting, models trained on reanalysis data, which has been corrected for modeling errors^43^, retain these corrections also at forecast time, displaying an improved skill at global medium-range weather forecasting^44–48^. In other words: these approaches outperform conventional atmospheric models as they suffer less from the problem needing to have the subgridscale-processes parameterized without having a perfect theory for this. For rainfall nowcasting, ML models improve accuracy^49–54^ by exploiting one of their key advantages: they can easily handle observational data such as rainfall radar, which contains information about processes beyond what is resolved in numerical schemes.

Similar evidence exists beyond just weather forecasting. Flood forecasting suffers from many unresolved processes in the hydrological modeling, making conventional forecasting hard and thus favoring ML^55,56^. Wildfire risk prediction requires linking earth observation with anthropogenic and meteorological drivers, thus benefiting from the flexibility of ML^57^. Seasonal ENSO^58–61^ and derived drought forecasting^22^ has always relied on empirical analysis of statistical patterns, which ML excels in. Many of these success stories have only been possible through adapting large deep neural networks which have been optimized to handle high-dimensional data (such as 3D fields), e.g., convolutional neural networks^62^ or transformers^63^.

### Moving from hazards to impacts

So far, most efforts to use ML have focused on hazard forecasts and have yet to trickle down the early warning chain. A critical next step is impact-centric forecasting. Here, ML can make use of predictors previously unavailable: it can leverage any predictive inputs to produce the best-possible forecast. Conventional approaches are often too rigid to incorporate such information. A central approach here is using weather forecasts as predictors and an impact-related quantity as target. In other words, the ML model maps from weather to impact, and is trained by collecting datasets of this mapping in the past. For instance, the impact of weather extremes on vegetation status as observed from satellite imagery can be predicted^64–67^, leveraging the vast archive of satellite products and aligning it with past weather information to create training datasets. In addition to the weather forecasts, other inputs such as socioeconomic time series can be easily added – e.g., to forecast food insecurity and famine^68,69^.

Beyond just traditional meteorological, geospatial, and socioeconomic data, deep neural networks can incorporate predictors such as social media posts, archived reports, or radio news^70^, which can be highly heterogeneous and are typically not considered in current EWS.

### Towards localized warnings

Warnings are most helpful, if they take the local context into account^71^. Already the push in accuracy and speed of ML models will help bring forecasts to higher resolution. For instance, recent AI models greatly reduce the tracking errors of tropical cyclones^45,46^. Beyond that, the move to impact-centric forecasting allows skipping scales and incorporating local information. For example, the link from weather to vegetation could be done using high-resolution satellite imagery^64,66,67^, which provides land-surface information available at field-scale (~10 m)^72^. In fact, beyond just satellite imagery, any local variable can be used to condition the forecast. E.g. this could be maps of the built-up infrastructure or the terrain for landslide risk modeling^73^.

Looking forward, text can potentially play a big role at localizing and personalizing warnings. For example, if privacy and ethical concerns are properly addressed, personal user data such as socioeconomic status, health information or available infrastructure can improve the contextualization of and increase trust in warnings. Here, the EWS for climate risk could become similar in spirit to the EWS that already exist in personalized medicine for disease risk^74^ – and thus, in similar ways benefit from ML models.

### Democratizing access globally

The quality of forecasts and warnings varies significantly across the globe, not merely due to differences in epistemic predictability between locations, but primarily because of disparities in data availability, which are largely influenced by a country’s economic conditions^75^. ML can accelerate the democratization of access to EWS. For instance, while conventional approaches often have skill-gaps in regions with low availability of primary data, ML can bridge the gaps with alternative data streams. Flood forecasting based on ML can provide skillful predictions in many un-gauged basins globally^76^. Here, also Earth observations play an important role. Many satellite observations are globally available, making them an interesting substitute for conventional data: e.g., an ML rainfall nowcasting model can be trained to forecast radar data globally, by only using satellite inputs^77^ and the Sentinel-1 & -2 satellites help mapping critical ecosystem variables^78^. On the other hand, many national hydrometeorological services (NHMS) struggle with insufficient observational infrastructure or limited modeling and forecasting systems or access to those^79,80^. Here, AI and ML may allow NHMS to overcome those challenges, e.g. because it can fill data gaps or due to more (cost-)efficient modeling — but only if open-source implementations are available, knowledge is shared and public institutions are supportive. The role of national hydrometeorological services and nationally mandated institutions for early warning (such as civil protection agencies) remains central to this democratization process. While private sector innovation contributes significantly to developing ML models and infrastructure, public institutions retain the local contextual knowledge and jurisdiction needed to align EWS with national disaster risk-reduction strategies.

The adoption of AI in EWS introduces macro-level and individual-level risks that demand critical attention. A key concern lies in the concentration of essential EWS infrastructure within a few entities, particularly private corporations. If governments become heavily reliant on proprietary AI platforms for monitoring, predicting, and alerting about natural hazards, this could create vulnerabilities to national security and public safety. Over-dependence on private infrastructure risks allowing external entities to influence or disrupt critical services during emergencies. To mitigate this, there is an urgent need for open-source AI models and systems, transparent standards, and international cooperation that ensures equitable access to these technologies.

Collaboration between the private sector, research institutions, and NHMS is needed to maximize the potential of ML for EWS while maintaining the necessary levels of accountability and public oversight. To truly democratize access, policies must foster partnerships that empower national entities without rendering them dependent on proprietary, inaccessible technology. Open standards, capacity-building initiatives, and institutional support are key elements to enabling NHMS and other nationally mandated institutions to fully leverage AI and ML, ensuring they can play a maximally beneficial role in protecting lives and livelihoods.

### Improve communication

Historically, a warning is a spoken or written message, that is, a one-way, relatively abstract mode of communication. Now ML allows to change that. More intuitive warnings are possible by creating photorealistic images of landscapes or properties affected by a predicted hazard with generative AI^81^. By including text-capability, ML models become chatbots, enabling new levels of communication. Chatbots are able to adapt messages to their users’ needs. Interactivity allows to display additional information upon interaction. Of course, this is also possible with conventional web-apps, but the degree of interactivity increases with the advent of large-scale AI. One important aspect of interactivity is explainability^82^, while Chatbots are not yet able to reliably explain why they came up with certain outcomes, a large body of work embraces methods to explain the outputs of deep neural networks and other ML models^83–85^. Integrating such techniques into warnings improves upon the situation with current conventional approaches: they are often too complex to easily decipher the underlying reasons for a prediction.

## Foundation models as a path forward

A technological path forward for AI-based EWS is the development of foundation models (FMs). A foundation model is typically a large deep neural network trained in a self-supervised manner on a large body of unlabeled data, to subsequently enable its application to many different (downstream) tasks. A first class of foundation models have been Large Language Models (LLMs)^86–89^, trained essentially on textual information, from Wikipedia, scientific papers and ArXiV to news, blogs and reports. These have been followed by Large Multi-Modal Models (LMMs)^90–93^, trained in addition on images and sometimes audio and video.

Recent works have introduced Foundation Models for Meteorology and for Geospatial applications^94–96^. The former are trained on large archives of atmospheric data, and can subsequently perform, e.g., weather forecasting^97^, climate projections or air quality forecasting^98^. For Meteorological FMs, the trend is to move from reanalysis-based training towards directly training on measured weather data, mainly from Earth Observation, as it represents most closely the real Earth system. The vast availability of satellite imagery is also exploited by geospatial FMs, e.g.,^99^, which are thus useful for any type of mapping task, e.g., land cover mapping or biomass estimation.

**Fig. 3:**
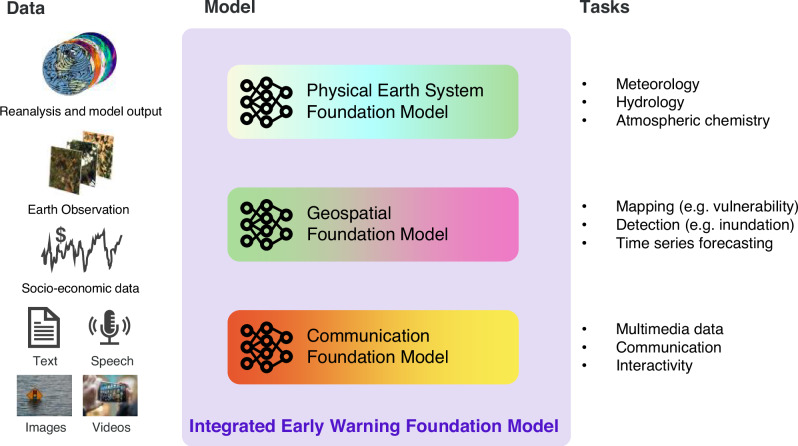
Vision for an Integrated Early Warning Foundation Model. Integration of currently developed foundation models into a modular Early Warning Foundation Model (center) allowing for ingestion of diverse data (left) and for addressing prediction and communication tasks (right).

For localized and impact-centric early warning, the combination of meteorological and geospatial data, as well as socioeconomic data appears necessary. It is not difficult to imagine a new generation of FMs, trained on all three such data streams, or instead the coupling of the separate FMs (Fig. 3). In fact, the techniques to achieve the bridging of different modalities have already been developed for LMMs^100–104^ and thus just need to be adapted for the high-dimensional data representing the whole Earth system.

A significant step in developing an early warning Foundation Model is incorporating capabilities similar to LMMs, such as processing natural language and images. Integrating text can unlock new predictors, personalized communication, and democratized access: the state-of-the-art is reviewed more comprehensively in the supplementary information. However, numerous challenges need to be addressed, which we discuss in the following sections. First, we address machine learning challenges related to biases, generalization and explainability, and then take a broader perspective on responsible AI in the context of Early Warnings for all.

## Towards robust AI-enabled EWS

### Biases and distribution shifts

One of the major advantages of machine learning early warning systems is that they can implicitly learn from large amounts of data, which is even more true for modern data-hungry foundation models. That is, we hand over control of the modeling assumptions (e.g., locality or recurrence) to the model itself. This, however, is no silver bullet to solve all problems – rather the usage of AI comes with its own challenges when these implicit modeling assumptions (so-called inductive biases^105^) are improper. Considering the impact of erroneous predictions, avoiding such failures to model the real world is crucial in an early warning foundation model.

One cause that can lead to such failure in neural networks is their tendency to favor simplicity^106,107^. What reminds of Occam’s razor can manifest in various forms, such as a preference for lower frequencies and simpler data characteristics^108^. Ultimately, this simplicity bias can even result in causally relevant aspects being disregarded in favor of simpler yet causally irrelevant properties^109^. Such shortcut biases are not limited to classical application areas of deep learning and have also been shown to be relevant in earth system analysis^110^. Thus, a central challenge is to connect FMs with novel attempts to estimate causality in geospatial settings^111^.

Since ML models only learn a mapping from inputs to outputs, they are also fundamentally limited to be applicable only in cases where the inputs and the input-to-output relationships are similar to the training data^109,112^. However, this condition might not be fulfilled for Earth-related datasets^110,113^, especially considering climate-related hazards, some of which simply did not occur in the past. For conventional ML models, one would typically tackle such a sampling bias two-fold: on the one hand, tweaking datasets and training algorithms, such that all applications can be captured by the training data domain, and on the other hand, through uncertainty quantification: that is, explicitly labeling when the model may not be trusted.

For foundation models, their design characteristics already provide an important step towards robustness against distribution shifts. Typically, they are not trained to specifically solve the thought-after downstream task, but rather, on a generic self-supervised task like gap-filling or next-step prediction. Yet, FMs are trained at a much larger scale on diverse data, which should provide improved regularization and help with covering well the covariate domain. Also, related to climate change, an FM would have to be trained on many different local climatic conditions around the globe, which may allow for space-for-time substitution^114^ and climate analogs^115^ to mitigate the issue partly: an extreme event may be out-of-distribution at one location, but the norm in another. Still, further research is needed, in particular to aid with overconfidence and hallucination^116^ in generative FMs and allow for calibrated output uncertainties.

### Open data

Key datasets need to be available and harmonized in order for FMs to become feasible. In meteorology and more general hazard prediction, this has already been partly achieved^117–119^. For geospatial data, which is heavily dependent on data pre-processing (e.g., cloud masking, atmospheric corrections), there are some initiatives^120–122^, but no consensus has evolved yet. In both cases, publicly funded data from continuous observatories, satellites, and radar are commonly available and provide relatively global coverage. Further down the early warning chain, the situation becomes more dire: socioeconomic data is seldom available at finer resolution than district level and often not standardized across countries. Moreover, much data on impacts is buried in reports stored in the archives of national agencies and insurance companies, without public availability. The past has shown, ML research thrives, when clear benchmarks and targets are available – designing such with EWS in mind is an open area of research.

### Bridging across scales

Such benchmark datasets will stumble upon a hurdle that will likely require new method development: Designing globally applicable, personalized EWS requires bridging across many scales. While medium-range weather forecasting has a resolution of 10 s of KM, socioeconomic data may be at any granularity from individual households to large countries and geospatial data can be of high spatial (just a few meters) or high temporal (a few minutes) fidelity. How to align these different data streams, set points of reference and also just plainly manage the storage and compute necessary for such an endeavor needs to be studied. Even more so, since a split-apply-combine, which is commonly used for scaling up methods, may be inapplicable: teleconnection and cross-boundary interactions can be relevant. Here, transformers^123^ are likely a good starting point for the required advancements: they are suitable for modeling long-range connections, e.g., for wildfire risk^57^, and they can be used to model many different data streams in the same latent space^102,104^, even if the data comes with different sparsity levels, e.g., linking county-level agricultural data to satellite imagery for crop yield prediction^124^.

### Respecting causality and scientific laws

EWS are most useful, if integrated into the decision-making process. For instance, one could imagine an AI-based EWS to be used from within a situation room, informing stakeholders about the current situation, relevant context and potential courses of action. Especially for the latter one, the system would need to be suitable for what-if analysis, i.e., run counterfactual scenarios. Such scenarios are only usable if the AI respects the causal mechanisms. Conventional ML can be extended with prior knowledge to obey causality in certain ways^125,126^, e.g., this has been recently used to study human displacement^127^. For FMs, how to achieve this is an active area of research: causal representation learning^128^. FMs for EWS ideally leverage causal representations, and at the same time provide a great test-bed for developing such methods: current mechanistic EWS can be used as ground-truth in emulation studies^129^, before moving to observational data. In addition, knowledge-guided machine learning (KGML) offers a promising pathway to enhance AI-based EWS by embedding domain expertise directly into the learning process^130^. This approach incorporates scientific principles and established rules to ensure that the system’s predictions and recommendations align with known laws of physics, economics, or other relevant fields. Hybrid modeling systems, which combine data-driven AI models with traditional mechanistic models, are particularly well-suited for these tasks^131^. Fine-tuning foundational models (FMs) within such hybrid frameworks poses a useful challenge, as it requires integrating large-scale, pre-trained models with the precise constraints and nuances of domain-specific knowledge. This fine-tuning can enhance the robustness and interpretability of EWS, allowing them to not only predict outcomes but also offer explainable insights grounded in established scientific and causal mechanisms.

## EWS that work for all

Apart from the above algorithmic challenges, there are related but broader concerns with making EWS work for all, providing access also for the Global South and disadvantaged communities, and avoiding power abuse. Some of these challenges implicitly exist also with traditional EWS. These challenges directly relate to the four pillars of early warning systems identified by the WMO^132^: risk knowledge, monitoring and forecasting, dissemination, and response capability (cf. Fig. 1). Ensuring equal access to AI-based EWS supports risk knowledge and monitoring across diverse contexts, while effective dissemination and community-responsive design are essential to foster actionable responses. With prudent design of an AI-based EWS there is the chance of resolving them in a favorable way, but this requires intensive future research.

### Representation and trustworthiness

A major concern with AI-based methods is the presence of biases in the training datasets as detailed in the previous section. These biases are particularly evident in the geographical distribution of data, where for instance in-situ observations are more abundant in the Global North. While Earth observation data from space can provide global coverage, geostationary satellites, for example, offer the best data around the equator. For societal data related to local vulnerability, these geographical biases are often even more pronounced. In addition to geographical biases, there are documented biases concerning ethnicity, gender, age, and other features^133^. However, human decision-making is also prone to biases and prejudices, which tend to be exacerbated in stressful conditions or when rapid decisions are required^134^. Moreover, humans have a limited capacity to process and integrate vast amounts of knowledge and experience. This is where AI systems have the potential to help, by accessing more extensive information and transferring knowledge (e.g., from one location to another) while still accounting for local context—provided that (1) the AI has access to appropriate data, and (2) some level of generalization can be assumed.

Furthermore, an AI-based system can be carefully developed during calm times, making sure it maintains objectivity and follows norms and rules during emergencies, where its warnings will be independent of the subjective stress of the situation. Strides in this direction have already been made, e.g., with large language models where for instance echoing racist speech is being avoided^135^. Approaches towards this Fair Learning are currently intensively researched with Reinforcement-Learning-based approaches. Yet, this reveals the important question of who is making the respective design decisions. It is evident that the beneficiaries from such systems should have agency in the design loop. This should both increase the objective quality of warnings, taking local conditions into account, and the trustworthiness, which is a function of the reliability of the system and the psychological identification of the humans working with it^136^. It is less clear how this can be achieved and the level at which each decision should be made (community, district, country, …). Research will be needed to scrutinize respective tradeoffs, which also relate to economic and infrastructural capabilities that are required to adequately deploy and work with such an EWS.

### Ownership and agency

Data and tools provide power, raising the question about their ownership, especially to address the potential risks associated with the centralization of power: when control over these resources is concentrated in the hands of a few, typically external entities, it can lead to power imbalances that disenfranchise local communities—those who are most affected by climate risks. Ensuring data ownership and agency for local communities is therefore essential. This involves not only making data accessible but also participatory approaches to data collection, interpretation, and usage^137^. Such empowerment helps to tailor solutions that are culturally relevant and more effective at addressing local needs. Furthermore, it supports sustainable practices and builds resilience by fostering local expertise and leadership in climate-adaptation strategies. Thus, research is needed on how to effectively implement community-driven governance models for data ownership in climate-adaptation strategies, e.g., through data trusts (cf.^138^).

### Addressing the digital divide

Another prerequisite for effective AI-based EWS is addressing the digital divide: While advancements in technology have somewhat mitigated the accessibility issues related to digital-based early warning systems, particularly in urban and semi-urban areas, significant challenges persist in rural and underdeveloped regions where infrastructure remains inadequate. As global connectivity improves, the focus increasingly shifts towards affordability and literacy — data access costs need to be affordable and content understandable. Factors such as educational level and age need to be considered. Ensuring that EWS are not only physically accessible but also economically viable and user-friendly will thus be crucial.

At least, in terms of energy and computational costs it is likely that AI-based EWSs can be “cheaper” than traditional ones. For instance, current efforts on medium-range weather forecasting allow AI-based forecast with a small fraction of the classical computation, enabling inference on a laptop^45^. Moreover, recent works on multilingual language models^139,140^ hold promise to enable distributing warnings in diverse languages, which is particularly important in the global south^133^. Future research needs to study how to bring these promises also to FM-based EWS, making them economically viable and accessible across different socioeconomic and geographical landscapes, considering variations in infrastructure, age, educational levels, and technical proficiency.

### Personalized warnings

The debate around personalized AI-based warnings versus empowering individuals to understand their own risks is multifaceted and nuanced. Personalized warnings offer specific benefits, such as providing timely, relevant information tailored to the unique circumstances and risk profiles of individuals. By running a personalization step directly on a private smartphone, data does not need to be sent back to a central server. This approach can enhance privacy, reduce latency, and lessen bandwidth usage. Smartphones can collect environmental data (like location, motion, or even nearby sounds), which can then be analyzed locally to generate personalized risk assessments. For example, a smartphone could detect that it is in a flood-prone area during heavy rains and alert the user accordingly. Of course, higher levels of aggregation with less specificity, e.g., warnings tailored to a community, are important, too. These levels of specificity can improve responsiveness, enhance trust, and increase compliance, particularly during emergencies when quick decision-making is crucial. Furthermore, these systems can help to optimize resource use and mitigate alert fatigue among populations in safer areas by reducing unnecessary alerts. On the other hand, the increased specificity of personalized warnings presents not only challenges in validation but also raises ethical concerns regarding equity, fairness, and potential biases in the AI models that generate these warnings. Personalized systems may inadvertently lead to unequal access to critical information if individuals are excluded due to technological barriers, such as lack of smartphone access or connectivity. There is also the risk of over-reliance on AI-generated messages, which could lead to misplaced trust if the model makes erroneous assessments. Safeguarding mechanisms are essential to ensure that AI systems do not amplify existing social inequities or inadvertently marginalize vulnerable groups.

Moreover, transparency in how these AI models function is crucial to maintain trust. Users should understand the basis upon which warnings are personalized, and the underlying data must be ethically sourced and used with explicit consent. Misuse or unauthorized access to personal data is a significant risk, particularly when dealing with sensitive information that could reveal an individual’s location or behavior patterns. Therefore, safeguarding user privacy and ensuring robust data governance practices are paramount to prevent misuse. Effective frameworks for accountability are also needed. If governments or institutions base actions on personalized warnings, a clear chain of accountability should be established, detailing how decisions are made, who is responsible, and what safeguards are in place to prevent misuse or misinformation. Balancing the benefits of personalization with ethical considerations and risk mitigation is critical for the future of AI in early warning systems.

Still, when these concerns are addressed, personalized warnings can provide significant advantages to empowering individuals with the knowledge to assess their own risks. This approach promotes autonomy and enduring knowledge, enabling people to make informed decisions independently of technological aids, which is particularly valuable in scenarios where technology may fail or be inaccessible, such as emergencies. Education on personal risk also fosters broader societal benefits by increasing general awareness. However, environmental data’s complexity and the rapid pace at which conditions can change often exceed the average individual’s capacity to stay informed without assistance. Personalized AI warnings can alleviate cognitive overload by distilling complex information into actionable insights, tailored to the user’s individual capacity. Thus, future work is needed to ensure that the integration of personalized AI-based warnings does not harm initiatives that enhance individual understanding of environmental risks, thus helping both immediate responsiveness and long-term resilience. Moreover, in doing so, it is necessary to ensure that the privacy and integrity of user data is preserved.

### Beyond immediate crisis response

**Fig. 4:**
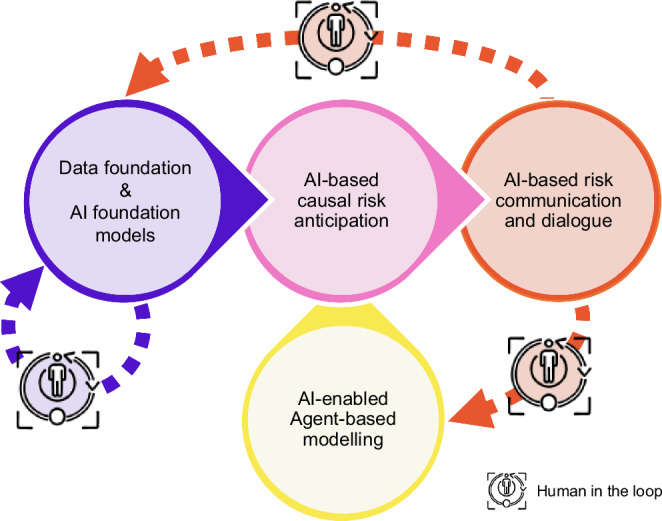
Integrative, artificial intelligence (AI) - enabled strategy for Early Warning of complex climate risks including an interactive component. The Early Warning FM leads to improved causal and data-informed risk anticipation, followed by AI-based communication. Anticipating disaster response as a risk factor^39^ should be addressed via Agent-based modeling embracing AI for parameter estimations. Information from the user-interaction should feed back to the model improvement.

The success of early warning systems (EWSs) also hinges on their ability to incorporate the response, the societal feedback, as well as considering long-term systemic impacts as risk factors beyond the classical hazard-exposure-vulnerability paradigm^39^. Traditional EWSs often focus on immediate responses to crises, but future EWSs must evolve to anticipate and mitigate long-term consequences, especially those arising from potentially misguided or counterproductive responses. Addressing these challenges involves exploring largely untested approaches. Integrating agent-based models can simulate societal behaviors for better prediction accuracy, while machine learning-based inverse inference reveals hidden systemic risks by tracing outcomes back to their root causes (Fig. 4). Gamification provides an innovative way to engage the public in data collection, not only enriching datasets for more accurate long-term predictions but also empowering individuals by involving them actively in the process, enhancing their awareness and preparedness.

Last but not least, current early warning systems, which are based on impacts caused by concrete weather conditions in the next hours to weeks, should be complemented by decadal time-scale early warning systems. Developing a decadal time-scale EWS for climate and weather risks is essential due to the increasing variability and extremity of weather patterns caused by climate change. Decadal EWS should guide effective adaptation measures, more targeted than what can be inferred from general climate change metrics and a general precautionary principle. This involves identifying vulnerable regions and sectors, planning infrastructure developments, and formulating policies that are resilient to long-term climatic changes. Effective communication strategies are needed to convey long-term risks and adaptations to governments, businesses, and communities, ensuring preparedness. Of course, reliable forecasts are a prerequisite, too. Hence, generally similar challenges as the ones mentioned above for short-term EWS need to be addressed, yet with an important addition: For this important challenge probabilistic and ensemble forecasts are highly relevant. These forecasts present a range of possible outcomes with associated probabilities, offering a more nuanced understanding of long-term risks and allowing the development and discussion of scenario components.

Additionally, integrating EWSs with policy-making is crucial for influencing long-term planning, ensuring decisions are guided by potential future impacts. Finally, fostering global collaboration among technical, community, and policy stakeholders is essential for developing innovative and resilient EWSs. Resulting key research questions are synthesized in Box 1.

Box 1 Key research questions to be addressed towards successful end-to-end Early Warning Systems (EWS)

1. How can causal structure and other forms of domain knowledge be incorporated into AI-based EWS to enhance the robustness of predictions while ensuring alignment with established geophysical processes?
2. What advancements are needed in AI models to accurately predict the occurrence, severity, and cascading impacts of extreme events, particularly in the context of increasing climate variability and non-linear interactions between hazards?
3. What frameworks and methods can be developed to effectively harmonize and align multi-scale, multi-modal data (e.g., socioeconomic, geospatial, meteorological) for global EWS applications without compromising on local specificity?
4. In the context of equitable access to EWS, how can open-source models, data ownership, and participatory design approaches be used to mitigate the risks associated with centralization of data and power imbalances in vulnerable communities and globally?
5. What strategies can be employed to enhance transparency, fairness, and accountability in AI-driven EWS, particularly in developing trust, avoiding biases, and ensuring ethical use of personalized risk information?
6. How can AI-based EWS be designed to facilitate effective decision-making by integrating uncertainty quantification, probabilistic modeling, and counterfactual analysis, thus allowing scenario-based planning for both immediate and long-term risk management?
7. How can we foster interdisciplinary research and collaboration to ensure that the design of AI-based EWS is informed by the real needs of stakeholders such as community members, policymakers, and humanitarian organizations, while also pushing the boundaries of AI technologies?

## Outlook

Integrated AI will lead to paradigm shifts in EWS. First, AI, especially Meteorological FMs, are already revolutionizing weather and hazard forecasts, leading to enormous improvements in lead times and resolution of warnings before disaster strikes. Second, multi-modal AI, materialized through Impact FMs, can leverage geospatial and socioeconomic data to assess vulnerabilities and tear down previously existing silos impeding effective impact-based warnings. Yet, a foundation model addressing the challenges mentioned above needs to resolve the dichotomy of representing generalizable global knowledge while accounting for local context. Society could reach large benefits from such a system, or rather, from such systems: the path towards integrated AI in the early warning chain is not a monopoly. Instead, modularity is key: ensuring checkpoints regarding hazards, direct and indirect impacts along the warning chain for traceability and diversity in modeling approaches, to achieve robustness and avoid concentration of power. Foundation models tend to follow such an approach, where task-specific fine-tuning follows a generic pre-training approach. It may be fine to have a few fundamental approaches to the latter, to save upon computational costs, while stakeholders can bring their own data for the former. For the societal integration of an AI-based EWS, it is essential to prioritize human-centric approaches in the system’s design and implementation. AI researchers need to interact with stakeholders such as humanitarians, economists, or sociologists, as well as the communities that shall benefit from the systems. Then, Integrated AI will lead to paradigm shifts in EWS. Especially for multi-hazard EWS, soon to be implemented across the globe for the UN Early Warnings for All initiative, AI has a lot to offer. Multi-modal AI can accelerate the shift from hazards to impacts, increase the locality and personalization of warnings, boost their accuracy and lead time, advance democratized access and enable a previously unseen level of interactivity. With such capabilities, our society’s ability to protect livelihoods from complex climate risk will be strengthened.

## Supplementary information

Supplementary Information

## References

[1] Pal, I., Shaw, R., Shrestha, S., Djalante, R. & Cavuilati, R. A. W. C. in *Toward Sustainable Development: Risk-informed and Disaster-resilient Development in Asia* 1–20 (Elsevier, 2021).

[2] UNDRR & WMO. in *Global Status of Multi-hazard Early Warning Systems: Target G* 1–27 (UNDRR, WMO, 2022).

[3] Kelman, I. & Glantz, M. H. in *Reducing Disaster: Early Warning Systems For Climate Change* (ed Ashbindu Zommers Singh, Z.) Ch. 5 (Springer Dordrecht, 2014).

[4] Golding, B. *Towards the “Perfect” Weather Warning* 1st edn (Springer Cham, 2022). **This book collects the state-of-the-art in EWS for weather-related fast-onset disasters without ML**.

[5] WMO. Cyclone Amphan highlights the value of multi-hazard early warnings. https://public.wmo.int/en/media/news/cyclone-amphan-highlights-value-of-multi-hazard-early-warnings (2020).

[6] Fakhruddin, S. H. M. *Recovery from the Indian Ocean Tsunami* 1st edn 59–72 (Springer Tokyo, 2015).

[7] Fujinawa, Y. & Noda, Y. Japan’s earthquake early warning system on 11 March 2011: performance, shortcomings, and changes. *Earthq. Spectra* **29**, 341–368 (2013).

[8] Garcia, C. & Fearnley, C. J. Evaluating critical links in early warning systems for natural hazards. *Environ. Hazards* **11**, 123–137 (2012).

[9] Rentschler, J. et al. Global evidence of rapid urban growth in flood zones since 1985. *Nature* **622**, 87–92 (2023). 37794266 10.1038/s41586-023-06468-9

[10] UNDRR. *Global Assessment Report on Disaster Risk Reduction* (United Nations Office for Disaster Risk Reduction (UNDRR), 2019).

[11] Thieken, A. H. et al. Performance of the flood warning system in Germany in July 2021 – insights from affected residents. *EGUsphere* **2022**, 1–26 (2022).

[12] De Perez, E. C. et al. Learning from the past in moving to the future: invest in communication and response to weather early warnings to reduce death and damage. *Clim. Risk Manag.* **38**, 100461 (2022).

[13] Potter, S. H. et al. The influence of impact-based severe weather warnings on risk perceptions and intended protective actions. *Int. J. Disaster Risk Reduct.* **30**, 34–43 (2018).

[14] Potter, S., Harrison, S. & Kreft, P. The benefits and challenges of implementing impact-based severe weather warning systems: perspectives of weather, flood, and emergency management personnel. *Weather Clim. Soc.* **13**, 303–314 (2021).

[15] Chaves-Gonzalez, J. et al. Anticipatory action: lessons for the future. *Front. Clim.* **4**, 932336 (2022).

[16] Zommers, Z. & Alverson, K. *Resilience: The Science of Adaptation to Climate Change* (Elsevier, 2018).

[17] Šakić Trogrlić, R. et al. in *Towards the “Perfect” Weather Warning* Ch. 2 (Springer, 2022).

[18] Bauer, P., Thorpe, A. & Brunet, G. The quiet revolution of numerical weather prediction. *Nature* **525**, 47–55 (2015). 26333465 10.1038/nature14956

[19] Dunstone, N. et al. Windows of opportunity for predicting seasonal climate extremes highlighted by the Pakistan floods of 2022. *Nat. Commun.* **14**, 6544 (2023). 37848427 10.1038/s41467-023-42377-1 PMC10582174

[20] OCHA. After action review on the CERF anticipatory action for typhoons. https://reliefweb.int/attachments/af219dbc-97e3-4df2-9126-e0b30f0e4083/220422%20Report%20on%20the%20After%20Action%20Review%20for%20CERF%20Anticipatory%20Action.pdf (2022).

[21] Funk, C. & Shukla, S. *Drought Early Warning and Forecasting: Theory and Practice* (Elsevier, 2020). **This book collects the state-of-the-art in drought early warning without ML**.

[22] Funk, C. et al. Frequent but predictable droughts in East Africa driven by a Walker circulation intensification. *Earths Future* **11**, e2022EF003454 (2023).

[23] DWD. The weather in Germany 2021. https://www.dwd.de/EN/press/press_release/EN/2021/20210830_the_weather_in_germany_in_summer_2021_news.html (2021).

[24] Reichstein, M., Frank, D., Sillmann, J. & Sippel, S. in *Climate Extremes and Their Implications for Impact and Risk Assessment* (eds J Sillmann, S Sippel, & S Russo) 341–353 (Elsevier, 2020).

[25] Lloyd, C. T., Sorichetta, A. & Tatem, A. J. High resolution global gridded data for use in population studies. *Sci. Data* **4**, 1–17 (2017). 10.1038/sdata.2017.1 PMC5283062 28140386

[26] Jurgilevich, A., Räsänen, A., Groundstroem, F. & Juhola, S. A systematic review of dynamics in climate risk and vulnerability assessments. *Environ. Res. Lett.* **12**, 013002 (2017).

[27] Bouwer, R., Pasquini, L. & Baudoin, M.-A. Breaking down the silos: building resilience through cohesive and collaborative social networks. *Environ. Dev.* **39**, 100646 (2021).

[28] Krishna Prabhakar, S. V. R. Implications of regional droughts and transboundary drought risks on drought monitoring and early warning: a review. *Climate* **10**, 124 (2022).

[29] Jain, S. K. et al. A brief review of flood forecasting techniques and their applications. *Int. J. River Basin Manag.* **16**, 329–344 (2018).

[30] Wu, D. & Cui, Y. Disaster early warning and damage assessment analysis using social media data and geo-location information. *Decis. Support Syst.*10.1016/j.dss.2018.04.005 (2018).

[31] Sufri, S., Dwirahmadi, F., Phung, D. & Rutherford, S. A systematic review of community engagement (CE) in disaster early warning systems (EWSs). *Prog. Disaster Sci.* **5**, 100058 (2020).

[32] Fakhruddin, B., Clark, H., Robinson, L. & Hieber-Girardet, L. Should I stay or should I go now? Why risk communication is the critical component in disaster risk reduction. *Prog. Disaster Sci.* **8**, 100139 (2020). 34977532 10.1016/j.pdisas.2020.100139 PMC8714028

[33] IMC. Libya flooding: situation report #9. https://reliefweb.int/report/libya/libya-flooding-situation-report-9-november-21-2023 (2023).

[34] BBC. Storm Daniel lashes Greece. https://www.bbc.com/weather/av/66730977 (2023).

[35] Dalela, P. K., Saldhi, A., Bhave, P. & Tyagi, V. Common alerting protocol compliant emergency warning and alert system for legacy broadcasting networks. In *2020 IEEE Conference on Cognitive and Computational Aspects of Situation Management (CogSIMA)* 1–5 (IEEE, 2020).

[36] Kelman, I., Glantz, M. H., Singh, A. & Zommers, Z. *Reducing Disaster: Early Warning Systems For Climate Change* 89–108 (Springer Dordrecht, 2014).

[37] Macherera, M. & Chimbari, M. J. A review of studies on community based early warning systems. *Jàmbá* **8**, a206 (2016). 10.4102/jamba.v8i1.206 PMC6014131 29955285

[38] Yore, R., Fearnley, C., Fordham, M. & Kelman, I. *Designing Inclusive, Accessible Early Warning Systems: Good Practices and Entry Points* (GFDRR; UCL Warning Research Centre; World Bank, 2023).

[39] Simpson, N. P. et al. A framework for complex climate change risk assessment. *One Earth* **4**, 489–501 (2021).

[40] Enenkel, M., Dall, K., Huyck, C. K., McClain, S. N. & Bell, V. Monitoring, evaluation, accountability, and learning (MEAL) in anticipatory action—earth observation as a game changer. *Front. Clim.*10.3389/fclim.2022.923852 (2022).

[41] Goodfellow, I., Bengio, Y. & Courville, A. *Deep Learning* (MIT Press, 2016).

[42] Raschka, S., Liu, Y. H. & Mirjalili, V. *Machine Learning with PyTorch and Scikit-Learn: Develop Machine Learning and Deep Learning Models with Python* (Packt Publishing Ltd, 2022).

[43] Hersbach, H. et al. The ERA5 global reanalysis. *Q. J. R. Meteorol. Soc.* **146**, 1999–2049 (2020).

[44] Kochkov, D. et al. Neural general circulation models for weather and climate. *Nature* **632**, 1060–1066 (2024). 10.1038/s41586-024-07744-y PMC11357988 39039241

[45] Lam, R. et al. Learning skillful medium-range global weather forecasting. *Science* **382**, eadi2336 (2023). **This paper introduced GraphCast, the first deep neural network beating the state-of-the-art medium-range weather forecasting model IFS**. 10.1126/science.adi2336 37962497

[46] Bi, K. et al. Accurate medium-range global weather forecasting with 3D neural networks. *Nature* **619**, 533–538 (2023). 37407823 10.1038/s41586-023-06185-3 PMC10356604

[47] Kurth, T. et al. FourCastNet: Accelerating Global High-Resolution Weather Forecasting Using Adaptive Fourier Neural Operators. In *Proc. Platform for Advanced Scientific Computing Conference (PASC '23)* 1–11 (Association for Computing Machinery, 2023).

[48] Nguyen, T., Brandstetter, J., Kapoor, A., Gupta, J. K. & Grover, A. ClimaX: a foundation model for weather and climate. In *Proc. 40th International Conference on Machine Learning* 25904–25938 (PMLR, 2023).

[49] Ravuri, S. et al. Skilful precipitation nowcasting using deep generative models of radar. *Nature* **597**, 672–677 (2021). 34588668 10.1038/s41586-021-03854-z PMC8481123

[50] Gao, Z. et al. Earthformer: exploring space-time transformers for earth system forecasting. *Adv. Neural Inf. Process. Syst*. **35**, 25390–25403 (2022).

[51] Espeholt, L. et al. Deep learning for twelve hour precipitation forecasts. *Nat. Commun.* **13**, 5145 (2022). 36050311 10.1038/s41467-022-32483-x PMC9436943

[52] Asperti, A. et al. Precipitation nowcasting with generative diffusion models. *Appl. Intell*. **55**, 187 (2025).

[53] Shi, X. et al. Convolutional LSTM network: a machine learning approach for precipitation nowcasting. In *Advances in Neural Information Processing Systems* (Curran Associates, 2015).

[54] Zhang, Y. et al. Skilful nowcasting of extreme precipitation with NowcastNet. *Nature* **619**, 526–532 (2023). 37407824 10.1038/s41586-023-06184-4 PMC10356617

[55] Kratzert, F. et al. Towards learning universal, regional, and local hydrological behaviors via machine learning applied to large-sample datasets. *Hydrol. Earth Syst. Sci.* **23**, 5089–5110 (2019).

[56] Nearing, G. et al. Global prediction of extreme floods in ungauged watersheds. *Nature* **627**, 559–563 (2024). 10.1038/s41586-024-07145-1 PMC10954541 38509278

[57] Prapas, I. et al. TeleViT: teleconnection-driven transformers improve subseasonal to seasonal wildfire forecasting. In *2023 IEEE/CVF International Conference on Computer Vision Workshops (ICCVW)* 3756–3761 (IEEE, 2023). **This paper introduced TeleViT, a transformer model that is able to leverage teleconnections to improve wildfire early warning**.

[58] Ham, Y.-G., Kim, J.-H. & Luo, J.-J. Deep learning for multi-year ENSO forecasts. *Nature* **573**, 568–572 (2019). 31534218 10.1038/s41586-019-1559-7

[59] Petersik, P. J. & Dijkstra, H. A. Probabilistic forecasting of El Niño using neural network models. *Geophys. Res. Lett.* **47**, e2019GL086423 (2020).

[60] Dijkstra, H., Petersik, P., Hernández-García, E. & López, C. The application of machine learning techniques to improve El Niño prediction skill. *Front. Phys*. 10.3389/fphy.2019.00153 (2019).

[61] Cachay, S. R. et al. The world as a graph: improving El Ni\~no forecasts with graph neural networks. Preprint at 10.48550/arXiv.2104.05089 (2021).

[62] Krizhevsky, A., Sutskever, I. & Hinton, G. E. Imagenet classification with deep convolutional neural networks. In *Advances in Neural Information Processing Systems* (Curran Associates, 2012).

[63] Dosovitskiy, A. et al. An image is worth 16x16 words:transformers for image recognition at scale. In *International Conference on Learning Representations* (ICLR, 2020).

[64] Benson, V. et al. Multi-modal learning for geospatial vegetation forecasting. In *Proc. IEEE/CVF Conference on Computer Vision and Pattern Recognition* 27788–27799 (IEEE, 2024).

[65] Barrett, A. B. et al. Forecasting vegetation condition for drought early warning systems in pastoral communities in Kenya. *Remote Sens. Environ.* **248**, 111886 (2020).

[66] Robin, C. et al. Learning to forecast vegetation greenness at fine resolution over Africa with ConvLSTMs. Preprint at https://arxiv.org/abs/2210.13648 (2022).

[67] Requena-Mesa, C., Benson, V., Denzler, J., Runge, J. & Reichstein, M. EarthNet2021: A large-scale dataset and challenge for Earth surface forecasting as a guided video prediction task. IEEE/CVF Conference on Computer Vision and Pattern Recognition Workshops (CVPRW, Nashville, TN, USA) 10.1109/cvprw53098.2021.00124 (2021).

[68] Martini, G. et al. Machine learning can guide food security efforts when primary data are not available. *Nat. Food* **3**, 716–728 (2022). **This paper introduced the models behind the WFP HungerMap, the first fully ML-based vulnerability assessment of food insecurity**. 37118143 10.1038/s43016-022-00587-8

[69] Foini, P., Tizzoni, M., Martini, G., Paolotti, D. & Omodei, E. On the forecastability of food insecurity. *Sci. Rep.* **13**, 2793 (2023). 36928341 10.1038/s41598-023-29700-y PMC10038988

[70] Luccioni, A. S., Pham, K. H., Lam, C. S. N., Aylett-Bullock, J. & Luengo-Oroz, M. Ensuring the Inclusive Use of NLP in the Global Response to COVID-19. in *Machine Learning and Principles and Practice of Knowledge Discovery in Databases. ECML PKDD 2021. Communications in Computer and Information Science* (eds Kamp, M. et al.) 259–266 (Springer International Publishing) (2021).

[71] Percy, F., Lourdes, M. & Macasil, K. Guidance document on people-centered risk-informed early warning systems. **48**, https://www.climatecentre.org/wp-content/uploads/CREWS_Guidelines_EWS_en.pdf (2023).

[72] Louis, J. et al. in *ESA Living Planet Symposium 2016* (ed. Ouwehand, L.) 1–8 (Spacebooks Online, 2016).

[73] Tehrani, F. S., Calvello, M., Liu, Z., Zhang, L. & Lacasse, S. Machine learning and landslide studies: recent advances and applications. *Nat. Hazards* **114**, 1197–1245 (2022).

[74] Goetz, L. H. & Schork, N. J. Personalized medicine: motivation, challenges, and progress. *Fertil. Steril.* **109**, 952–963 (2018). 29935653 10.1016/j.fertnstert.2018.05.006 PMC6366451

[75] Linsenmeier, M. & Shrader, J. G. Global inequalities in weather forecasts. Center for Open Science. https://ideas.repec.org/p/osf/socarx/7e2jf.html (2023). **You might wonder how this can be true when the ECMWF forecasts have so quickly improved in both the Northern and Southern Hemisphere. This is because most countries need to develop their own forecasts to get more high-resolution predictions**.

[76] Nevo, S. et al. Flood forecasting with machine learning models in an operational framework. *Hydrol. Earth Syst. Sci.* **26**, 4013–4032 (2022). **This paper introduced the Google FloodHub, which is the first global initiative leveraging machine learning for an operational EWS**.

[77] Hilburn, K. A., Ebert-Uphoff, I. & Miller, S. D. Development and interpretation of a neural-network-based synthetic radar reflectivity estimator using GOES-R satellite observations. *J. Appl. Meteorol. Clim.* **60**, 3–21 (2020).

[78] Mao, H., Kathuria, D., Duffield, N. & Mohanty, B. P. Gap filling of high‐resolution soil moisture for SMAP/Sentinel‐1: a two‐layer machine learning‐based framework. *Water Resour. Res.* **55**, 6986–7009 (2019).

[79] Gudoshava, M. et al. Advances, gaps and way forward in provision of climate services over the Greater Horn of Africa. *Front. Clim.* **6**, 1307535 (2024).

[80] World Meteorological Organization – Alliance for Hydromet Development. Hydromet gap report. https://library.wmo.int/idurl/4/68926 (2024).

[81] Luccioni, A., Schmidt, V., Vardanyan, V. & Bengio, Y. Using artificial intelligence to visualize the impacts of climate change. *IEEE Computer Graph. Appl.* **41**, 8–14 (2021). 10.1109/MCG.2020.3025425 33444127

[82] Vinuesa, R. & Sirmacek, B. Interpretable deep-learning models to help achieve the Sustainable Development Goals. *Nat. Mach. Intell.* **3**, 926–926 (2021).

[83] Lundberg, S. M. & Lee, S.-I. A unified approach to interpreting model predictions. In *Proc. 31st InternationalConference on Neural Information Processing Systems (NIPS'17)* 4768–4777 (Curran Associates, 2017).

[84] Samek, W., Montavon, G., Lapuschkin, S., Anders, C. J. & Müller, K.-R. Explaining deep neural networks and beyond: a review of methods and applications. *Proc. IEEE* **109**, 247–278 (2021).

[85] Ronco, M. et al. Exploring interactions between socioeconomic context and natural hazards on human population displacement. *Nat. Commun.* **14**, 8004 (2023). 38049446 10.1038/s41467-023-43809-8 PMC10695951

[86] Radford, A., Narasimhan, K., Salimans, T. & Sutskever, I. Improving language understanding by generative pre-training. Preprint at https://cdn.openai.com/research-covers/language-unsupervised/language_understanding_paper.pdf (2018).

[87] Touvron, H. et al. Llama 2: open foundation and fine-tuned chat models. Preprint at 10.48550/arXiv.2307.09288 (2023).

[88] OpenAI et al. GPT-4 technical report. Preprint at 10.48550/arXiv.2303.08774 (2023).

[89] Chowdhery, A. et al. PaLM: scaling language modeling with pathways. *J. Mach. Learn. Res.* **24**, 1–113 (2023).

[90] Radford, A. et al. Learning transferable visual models from natural language supervision. In *International Conference on Machine Learning* 8748–8763 (PMLR, 2021). **This paper introduced CLIP, the first image-text model doing multi-modal learning with a contrastive loss function**.

[91] Girdhar, R. et al. ImageBind one embedding space to bind them all. In *Proc. IEEE/CVF Conference on Computer Vision and Pattern Recognition* 15180–15190 (IEEE, 2023).

[92] Alayrac, J.-B. et al. Flamingo: a visual language model for few-shot learning. *Adv. Neural Inf. Process. Syst.* **35**, 23716–23736 (2022).

[93] Liu, H., Li, C., Wu, Q. & Lee, Y. J. Visual instruction tuning. In *Advances in Neural Information Processing Systems* 34892–34916 (NIPS, 2023).

[94] Jakubik, J. et al. Foundation models for generalist geospatial artificial intelligence. Preprint at https://arxiv.org/abs/2310.18660 (2023).

[95] Smith, M. J., Fleming, L. & Geach, J. E. EarthPT: a foundation model for Earth observation. Preprint at https://arxiv.org/abs/2309.07207 (2023).

[96] Tseng, G., Zvonkov, I., Purohit, M., Rolnick, D. & Kerner, H. Lightweight, pre-trained transformers for remote sensing timeseries. Preprint at https://arxiv.org/abs/2304.14065 (2023).

[97] Lessig, C. et al. AtmoRep: a stochastic model of atmosphere dynamics using large scale representation learning. Preprint at https://arxiv.org/abs/2308.13280 (2023). **This paper introduces AtmoRep, a novel, task-independent stochastic model of atmospheric dynamics that employs large-scale representation learning to skillfully address diverse applications such as nowcasting, temporal interpolation, model correction, and counterfactual analysis**.

[98] Bodnar, C. et al. Aurora: a foundation model of the atmosphere. Preprint at https://arxiv.org/abs/2405.13063 (2024).

[99] Han, B., Zhang, S., Shi, X. & Reichstein, M. Bridging remote sensors with multisensor geospatial foundation models. In *Proc. IEEE/CVF Conference on Computer Vision and Pattern Recognition* 27852–27862 (IEEE, 2024).

[100] Perez, E., Strub, F., Vries, H. d., Dumoulin, V. & Courville, A. FiLM: visual reasoning with a general conditioning layer. *Proc. AAAI Conf. Artif. Intell.*10.1609/aaai.v32i1.11671 (2018).

[101] Zhang, Y. & Yan, J. Crossformer: transformer utilizing cross-dimension dependency for multivariate time series forecasting. In *The Eleventh International Conference on Learning Representations* (ICLR, 2023).

[102] Mizrahi, D. et al. 4m: Massively multimodal masked modeling. In *Advances in Neural Information Processing Systems* 58363–58408 (NIPS, 2023).

[103] Rombach, R., Blattmann, A., Lorenz, D., Esser, P. & Ommer, B. High-resolution image synthesis with latent diffusion models. In *Proc. IEEE/CVF Conference on Computer Vision and Pattern Recognition* 10684–10695 (IEEE, 2022).

[104] Jaegle, A. et al. Perceiver IO: a general architecture for structured inputs & outputs. In *ICLR 2022**Conference* (ICLR, 2022).

[105] Goyal, A. & Bengio, Y. Inductive biases for deep learning of higher-level cognition. *Proc. R. Soc. A* **478**, 20210068 (2022).

[106] Teney, D., Abbasnejad, E., Lucey, S. & Van den Hengel, A. Evading the simplicity bias: training a diverse set of models discovers solutions with superior OOD generalization. In *Proc. IEEE/CVF Conference on Computer Vision and Pattern Recognition* 16740–16751 (IEEE, 2022).

[107] Shah, H., Tamuly, K., Raghunathan, A., Jain, P. & Netrapalli, P. The pitfalls of simplicity bias in neural networks. *Adv. Neural Inf. Process. Syst.* **33**, 9573–9585 (2020).

[108] Ackaah-Gyasi, K. N., Valdez, S., Gao, Y. & Zhang, L. Exploring spectral bias in time series long sequence forecasting. Preprint at https://kdd.org/kdd2023/wp-content/uploads/2023/08/ackaah-gyasi2023exploring.pdf (2023).

[109] Geirhos, R. et al. Shortcut learning in deep neural networks. *Nat. Mach. Intell.* **2**, 665–673 (2020). **This paper introduced the concept of shortcut learning which need to be tackled through careful evaluation for deep neural networks to generalize well**.

[110] McGovern, A., Ebert-Uphoff, I., Gagne, D. J. & Bostrom, A. Why we need to focus on developing ethical, responsible, and trustworthy artificial intelligence approaches for environmental science. *Environ. Data Sci.* **1**, e6 (2022).

[111] Nachtigall, F., Wagner, F., Berrill, P. & Creutzig, F. Built environment and travel: tackling non-linear residential self-selection with double machine learning. *Transport. Res. D: Transport Environ*. **140**, 104593 (2025).

[112] Schneider, S. et al. Improving robustness against common corruptions by covariate shift adaptation. *Adv. Neural Inf. Process. Syst.* **33**, 11539–11551 (2020).

[113] Meyer, H. & Pebesma, E. Machine learning-based global maps of ecological variables and the challenge of assessing them. *Nat. Commun.* **13**, 1–4 (2022). 35459230 10.1038/s41467-022-29838-9 PMC9033849

[114] Pickett, S. T. A. in *Long-Term Studies in Ecology: Approaches and Alternatives* (ed. Likens, G. E.) 110–135 (Springer New York, 1989).

[115] Hallegatte, S., Hourcade, J.-C. & Ambrosi, P. Using climate analogues for assessing climate change economic impacts in urban areas. *Clim. Chang.* **82**, 47–60 (2007).

[116] Ji, Z. et al. Survey of hallucination in natural language generation. *ACM Comput. Surv.* **55**, 1–38 (2023).

[117] Kondylatos, S., Prapas, I., Camps-Valls, G. & Papoutsis, I. Mesogeos: a multi-purpose dataset for data-driven wildfire modeling in the Mediterranean. In *Advances in Neural Information Processing Systems* (Curran Associates, 2024).

[118] Rasp, S. et al. WeatherBench 2: a benchmark for the next generation of data‐driven global weather models. *J. Adv. Modeling Earth Syst.* **16**, e2023MS004019 (2024).

[119] Kratzert, F. et al. Caravan-a global community dataset for large-sample hydrology. *Sci. Data* **10**, 61 (2023). 36717577 10.1038/s41597-023-01975-w PMC9887008

[120] Kuglitsch, M. M. et al. When it comes to Earth observations in AI for disaster risk reduction, is it feast or famine? A topical review. *Environ. Res. Lett.* **18**, 093004 (2023).

[121] Lacoste, A. et al. Geo-bench: toward foundation models for earth monitoring. In *Advances in Neural Information Processing Systems* (Curran Associates, 2024).

[122] Mendieta, M., Han, B., Shi, X., Zhu, Y. & Chen, C. Towards geospatial foundation models via continual pretraining. In *Proc. IEEE/CVF International Conference on Computer Vision*. 16760–16770 (IEEE, 2023).

[123] Vaswani, A. Attention is all you need. In *Advances in Neural Information Processing Systems* (Curran Associates, 2017).

[124] Lin, F. et al. MMST-ViT: Climate change-aware crop yield prediction via multi-modal spatial-temporal vision transformer. In *Proc. IEEE/CVF International Conference on Computer Vision (ICCV)* 5751–5761 (IEEE, 2023).

[125] Runge, J. et al. Inferring causation from time series in Earth system sciences. *Nat. Commun.* **10**, 2553 (2019). 31201306 10.1038/s41467-019-10105-3 PMC6572812

[126] Rothenhäusler, D., Meinshausen, N., Bühlmann, P. & Peters, J. Anchor regression: heterogeneous data meet causality. *J. R. Stat. Soc. Ser. B Stat. Methodol.* **83**, 215–246 (2021).

[127] Tárraga Habas, J. M., Sevillano Marco, E. & Miranda, M. T. *The State-of-the-Art on Drought Displacement Modelling* (iDMC Internal Displacement Monitoring Centre, 2022).

[128] Schölkopf, B. et al. Toward causal representation learning. *Proc. IEEE* **109**, 612–634 (2021). **This paper introduced Causal Representation Learning, a new paradigm for machine learning, where the latent space is not solely based on correlations, but on causation**.

[129] Kekić, A. et al. Evaluating vaccine allocation strategies using simulation-assisted causal modeling. *Patterns* **4**, 100739 (2023). 37304758 10.1016/j.patter.2023.100739 PMC10155501

[130] Karpatne, A., Kannan, R. & Kumar, V. *Knowledge Guided Machine Learning: Accelerating Discovery using Scientific Knowledge and Data* (CRC Press, 2022).

[131] Reichstein, M. et al. Deep learning and process understanding for data-driven Earth system science. *Nature* **566**, 195–204 (2019). 30760912 10.1038/s41586-019-0912-1

[132] WMO. *Early Warnings for All: Executive Action Plan 2023-2027* (World Meteorological Organization, 2022).

[133] Naudé, W. & Vinuesa, R. Data deprivations, data gaps and digital divides: lessons from the COVID-19 pandemic. *Big Data Soc.* **8**, 20539517211025545 (2021).

[134] Svenson, O. & Maule, A. J. *Time Pressure and Stress in Human Judgment and Decision Making* (Springer Science & Business Media, 1993).

[135] Solaiman, I. & Dennison, C. Process for adapting language models to society (palms) with values-targeted datasets. *Adv. Neural Inf. Process. Syst.* **34**, 5861–5873 (2021).

[136] Bostrom, A. et al. Trust and trustworthy artificial intelligence: a research agenda for AI in the environmental sciences. *Risk Anal.* **44**, 1498–1513 (2024). **This work presents a research agenda emphasizing the importance of user engagement, co-development strategies, and insights from risk communication to enhance trust and trustworthiness in AI applications within environmental sciences**. 37939398 10.1111/risa.14245

[137] de Vos, A., Preiser, R. & Masterson, V. A. Participatory data collection. *The Routledge Handbook of Research Methods for Social-Ecological Systems*, Vol. 119 (Routledge, 2021).

[138] Creutzig, F. From smart city to digital urban commons: Institutional considerations for governing shared mobility data. *Environ. Res. Infrastruct. Sustain.* **1**, 025004 (2021).

[139] Ogueji, K., Zhu, Y. & Lin, J. Small data? No problem! Exploring the viability of pretrained multilingual language models for low-resourced languages. In *Proc. 1st Workshop on Multilingual Representation Learning* (eds Ataman, D. et al.) 116–126 (Association for Computational Linguistics, 2021).

[140] Wei, X. et al. PolyLM: an open source polyglot large language model. Preprint at 10.48550/arXiv.2307.06018 (2023).

