# Supplementary material for: Early warning of complex climate risk with integrated artificial intelligence
Source: Nat Commun. 2025 Mar 15;16:2564. doi: 10.1038/s41467-025-57640-w (PMC11910612; doi:10.1038/s41467-025-57640-w)
Supplement: Supplementary file 1 — Supplementary Information [file 41467_2025_57640_MOESM1_ESM.pdf]

Early warning of complex climate risk with integrated artificial intelligence

Supplementary Information

Markus Reichstein[1,2,3] Vitus Benson[2,3,4], Jan Blunk[5], Gustau Camps-Valls[6], Felix Creuzig[7,8], Carina Fearnley[9], Boran Han[1],Kai Kornhuber[10,11], Nasim Rahaman[12], Bernhard Schölkopf[12], José María Tárraga[6], Ricardo Vinuesa[13], Karen Dall[15], Joachim Denzler[2, 5], Dorothea Frank[3], Giulia Martini[15], Naomi Nganga[16], Danielle Maddix Robinson[1] , Kommy Weldemariam[1]

1 Amazon Web Services

2 ELLIS Unit Jena

3 Max-Planck-Institute for Biogeochemistry, Department of Biogeochemical Integration

4 ETH Zurich

5 University of Jena, Computer Vision Group

6 University of Valencia

7 Mercator Research Institute on Global Commons and Climate Change and Technical University Berlin

8 University of Sussex

9 University College London

10 Lamont-Doherty Earth Observatory, Columbia University, New York, USA

11 International Institute for Applied Systems Analysis (IIASA), Laxenburg, Austria

12 Max-Planck-Institute for Intelligent Systems

13 FLOW, Engineering Mechanics, KTH Royal Institute of Technology, Stockholm, Sweden

14 German Red Cross

15 World Food Program

16 Kenya Red Cross

Table S1: Overview of early warning short-comings for past events and how AI could help to address the short-comings in the future.

	**EUROPEAN FLOODS 2022**[1-3]	**HORN OF AFRICA DROUGHT 2016-2017 AND 2019FF**	**MIDWEST HEAT-FIRE-SMOKE DISASTER 2023**
**GENERAL SITUATION**	o July 2021 heavy rainfall in Germany, Belgium, France and UK [4]<br>o Flash floods afterwards [5]<br>o Biggest impact in small river Ahr: 196 dead	o Five failed rainfall seasons 2019-2023<br>o Devastating multi-year drought in Somalia, Ethiopia and Kenya<br>o Over 36 Mio people affected (__REF__ https://reliefweb.int/report/ethiopia/horn-africa-drought-regional-humanitarian-overview-call-action-revised-24-august-2022)	o Very warm and dry spring<br>o Wildfires in Canada [6]<br>o June 2023 smoke travels to midwest: record-shattering airpolution in NYC[7]<br>o Large impacts on Health[8]
**EXISTING EWS**	o EFAS european flood alerts were send out[9]<br>o Warning chain included neighboring counties and the media	o FEWSNet food security classification forecast based on ENSO forecasts<br>o SPI forecasts from ICPAC & VCI forecasts from RCMRD<br>o Anticipatory action based on those triggers + vulnerability data, e.g. by Kenya Red Cross on County level	o Airnow.gov airquality forecast for 2 days in advance<br>o Communication to public very late<br>o Measures taken were canceling of public events etc.
**OBSERVATIONS & WEATHER FORECAST**	Shortcomings:<br>River Gauges were damaged by the high water levels<br>Weather forecast was already quite good 2 days in advance, not the main limiting factor<br><br>Role of AI and challenges:<br>Global Meteorological FM offer orders of magnitude higher speed allowing larger ensembles and thus better probabilistic estimates.<br>Meteorological FM radar map forecasts are better at heavy rainfall events than weather models[14]<br>Video gauges (not in contact with the water) AI calibrated to predict water levels and flows may be more robust (minor point), but calibration needed	Shortcomings:<br>Lack of hydrometeorological stations in parts of the area (e.g. Somalia) => insufficient precipitation data<br>Biases in spatialized precipitation estimates (fusing station and satellite data)<br>Lack of food insecurity data, i.e. vulnerability<br>Because of longer lead times and the superposition of teleconnections (ENSO, IOD) forecasts are uncertain<br>Local-scale forecasts for districts or villages a major challenge<br>confidence levels are stated as one of the reasons the early decision making was delayed [23].<br>Because of inherent uncertainties, acting on seasonal forecasts is currently estimated to take up to a decade before a FbF system would generate value (due to false alarm cost etc)[12]<br><br>Role of AI and challenges:<br>Geospatial foundation models may lead to better interpolation and fusion of satellite and station data by maximising data input and via generalization and transfer from other areas. This may include new anti-causal learning approaches. For instance, precipitation can be inferred anti-causally from soil moisture observation.<br>Similarly, Martini et al.[10] showed that vulnerability can be estimated from proxy data, using a global machine learning-	Shortcomings:<br>Citizen science, especially cell phone data, is challenging to use in existing EWS.<br>Weather forecast necessary for circulation, but ultimately it is the transport of smoke, that is relevant for air quality. Thus need integration of weather forecast with fire forecast for air quality forecasting.<br><br>Role of AI and challenges:<br>AI-based methods to integrate sparse and low quality sensor data, e.g. human mobility patterns to estimate health effects.<br>AI-based airquality forecasts can leverage teleconnections —> longer lead times<br>Meteorological FMs may be extended to include Chemical Transport Modeling, which offers order of magnitude speed ups in simulation of the dynamics of wildfire smoke.

		based model trained on primary data outside the area of consideration.<br>Meteorological FMs may be extended towards both, seasonal forecast and local downscaling – research needed on both teleconnections and hyperlocal influence of soils and vegetation.	
**HAZARD & IMPACT FORECAST**	Shortcomings:<br>Flood levels were not precisely and locally enough predicted, because of insufficient resolution and negligence of debris flow and morpho-dynamic processes.<br>Impact forecast non-existent<br>Detailed forecasts for smaller river basins were missing<br><br>Role of AI and challenges:<br>Machine learning–accelerated computational fluid dynamics[11] can overcome computational limitations of hydro-morpho-dynamic modelling.<br>AI-based stream flow forecasts to capture local circumstances and debris flow & scale to ungauged basins<br>AI guided forensic analysis of exposure and vulnerability using multi-modal approaches, local fine-tuning of geospatial foundation models.<br>Limitations include knowledge of high-resolution morphology including bottlenecks such as bridges or channels and stochasticity of debris flow, and societal data for vulnerability assessment. Space-for-time generalization acceptable?	Shortcomings:<br>Current EWS are centered on weather only focus on single variables, e.g. rainfall, not compound or cascading events relatively coarse-grained information, struggling in low-information and high-risk areas like pastoralist regions during 2016 drought, timing of onset biased<br>Impacts such as vegetation or crop conditions not explicitly addressed, often based on simple hazard thresholds, not accounting for ecological and socio-economic conditions<br>Need for holistic multi-hazard approach, including complex cascading effects (drought, fire, flood, locust outbreaks)<br><br>Role of AI and challenges:<br>Localized impact forecasts help increase the efficiency of existing funding. Directly leveraging high resolution Earth observation to map impacts on vegetation and harvests conceivable, e.g. with the EarthNet models[12]. Text information successfully used as additional feature to predict food crises[13] including socio-economic conditions.<br>Language Foundation Models work without a notion of “scale”. For Hazard & Impact forecasting need to integrate data across many scales. Multi-modal transformer models like Perceiver IO[15] hint at one possible avenue, but range of scales treated there is limited compared to scales in Disaster Early Warning chain.<br>Impact foundation models can improve prediction skills by integrating, weather, land surface and socio-economic variables	Shortcomings:<br>Wildfire forecasts need to take into account memory effects, such as a previously very dry season, for fuel estimation.<br>Airquality forecasts suffer from a lack of integration of teleconnections. There was room for predictability from the wildfire season in Canada, which was not leveraged in the midwest, as airquality warnings had only been issued 2 days in advance.<br>PM2.5 values are still quite abstract for decision makers and the general public, hence could go one step further and make impact forecasts on health and economics.<br><br>Role of AI and challenges:<br>Wildfire modeling with recurrent neural networks and transformers to account for long-range memory effects<br>Transformers for leveraging teleconnections, such as the circulation-driven pattern<br>Impact foundation model for integrating weather -> socio-economic -> health<br>But: This requires branching across Silos
**WARNING COMMUNICATION, DECISION AND LONGER-TERM ADAPTATION**	Shortcomings:<br>Warnings were issued 1-2 days in advance, but with numbers (mm of rain, flood levels), which are not always effective with respect to making timely decisions[13]<br><br>Role of AI and challenges:<br>Conditioned on flood levels and digital elevation models, expected inundation areas and expected damages can be visualized with (generative) AI based maps and photo-realistic	Shortcomings:<br>Issues with respect to interpretation of warning levels regarding the food security situation because of their abstract and coarse nature, resulting in disagreements over the appropriate response and course of action, and even leading to delays in funding and disengagement of some donors<br>Status-quo bias in decision making with a tendency to resist action<br>Lack of accessibility and transparency in early warning reports, mainly available in English rather than local languages, using complex scientific jargon, and neglecting information sharing with drought-affected communities,	Shortcomings:<br>Silo thinking lead to communication of warning very late, and to top down decisions such as last minute closing of events.<br>Health impacts are often related to insufficient protection of vulnerable people, which could be improved by better communication.<br><br>Role of AI and challenges:<br>Early warning foundation model for communication to local decision makers, event organizers etc.

	representations. In addition, generation of language based (written or audio for visually impaired) warnings are indicated. ChatBot for interactivity in these communications.<br>Situation room: Early warning foundation model to enable local decisionmakers to have detailed access to relevant knowledge	limited their usefulness in helping vulnerable populations take necessary precautions<br>Quantifying the impact of the interventions missing<br>Identification of the root causes of vulnerability<br>Insufficient finance, especially 2021-2023<br>Whole region is considered “high alert”, but lack of granularity<br>Role of AI and challenges:<br>AI based forecasts of impacts (see above) allow more tangible, interpretable and fine-grained forecasts of food insecurity (and related issues like WASH or migration).<br>Causal analysis allows for estimating effectively measure cause-effect estimations and thresholds of impacts the effect of interventions (more research needed).<br>Causal representation learning to discover causal pathways and enable what-if modeling for planning & evaluation of anticipatory action<br>AI can assist in optimizing the dissemination of warnings based on factors like geographical location, population density, and vulnerability indices, ensuring that the right information reaches the right people at the right time.<br>Large-language models may efficiently translate warnings to natural language including local languages and inclusivity.<br>Early warning foundation models for informing front-line humanitarian workers in their local language —> enabling interactivity + democratization	Longer-term AI-based risk assessments (decadal early warning) to understand the distribution of heat-fire-smoke events in the future and enable adaptation, especially through better infrastructure and education

Supplementary text:

In-depth review of opportunities and challenges of Foundation modeling approaches for early warning

Machine learning methods, especially deep neural networks (DNNs), have demonstrated to successfully tackle important limitations of existing EWS. So far, these efforts have nevertheless mostly focused on hazard forecasts, and have yet to trickle down the whole early warning chain.

Weather forecasting with deep neural network emulators, known as **Meteorological Foundation Models (FMs)**, has gained traction in recent years. Trained on extensive historical observations using self-supervised learning, these models predict the next time step, ensuring fast and accurate numerical weather prediction (NWP). Existing meteorological FMs may be classified broadly into two categories: global medium-range forecasting models[14-18] trained on the ERA5 3D reanalysis dataset[19] and regional precipitation nowcasting models[20-22] [23-25] trained on satellite radar observations. Meteorological FMs outperform traditional approaches on small-scale phenomena related to storms and rainfall: GraphCast[26] and PanguWeather[16] greatly reduce the tracking errors of tropical cyclones and NowCastNet[25] is skillful also for extreme precipitation events, something which was previously considered intractable. Most meteorological FMs are built on top of approaches from Computer Vision[27]: Convolutional Neural Networks or Vision Transformers [28],and recently Graph Neural Networks[15,29] are also gaining traction.

Moving forward, meteorological FMs need now take on a probabilistic perspective, which can enable the generation of large ensembles and thereby of sharp worst-case scenarios useful for early warning. They might transition to utilizing raw multimodal observations, moving away from depending on existing data assimilation for a coherent reanalysis dataset. Additionally, meteorological FMs should place more emphasis on the challenging yet important subseasonal-to-seasonal time scale for predictability. So far, most work on seasonal forecasts with ML focuses on coarse indicators. Especially the ENSO phenomenon, which greatly influences agricultural weather in Africa and South America, can be predicted well with deep neural networks [30-33]. Such phenomena are crude descriptions of the actual dynamics unfolding in the Earth system [34], which is why moving to dense meteorological FMs is both promising and necessary [35].

On land, distinguishing between hazard and impact forecasts blurs. Consider the European Floods use case: traditional hydrological models for stream flow forecasts needed basin-specific tuning. However, treating stream flow forecasts as a time series, Long Short-

Term Neural Networks (LSTMs) trained on cross-basin data outperform previous methods[36,37]. Google FloodHub operationalizes this, providing flood forecasts in over 80 countries, offering early warnings to large populations[38].

Traditional EWSs issue warnings based on administrative boundaries, which may not align with the disaster's actual spatial pattern. AI enables dense spatio-temporal predictions, a fact that has exhibited important implications for example in the Horn of Africa drought use case, where humanitarians can now transition from district-level vegetation health forecasts[39] to using the EarthNet AI models [40-42], which provide predictions for individual fields and communities by using high-resolution satellite imagery. Further developments of such approaches may be called **Geospatial FMs**: They leverage the vast availability of satellite data through self-supervised learning. Here we posit that geospatial FMs, particularly those focusing on forecasting, can have a large impact on EWSs. For instance, like the EarthNet models [40-42], the EarthPT foundation model[43] can out-of-the-box predict vegetation greenness indices. Hence, this allows for highly targeted triggers for anticipatory action. Similarly, wildfire risk follows localized patterns, yet current risk maps are typically provided at regional level, a fact that has been heavily criticized for instance in Greece[44]. Now, deep learning-based approaches can deliver spatially explicit maps of wildfire danger[45]. The upcoming geospatial FMs, like the Prithvi FM[46] for burned area segmentation and the Presto FM[47] for fuel moisture estimation, show promise for prediction. However, the application of generative AI for dense spatio-temporal prediction remains under explored. As lead time increases, uncertainty in drought forecasting and wildfire risk estimation grows, and diffusion models[48] may offer sharp and plausible predictions, avoiding unphysical mean predictions.

Socio-economic variables, which are crucial for understanding vulnerabilities and impacts, are often limited to coarse administrative levels and infrequent sampling intervals. Nevertheless, ML can successfully leverage them to predict drought impacts in the Horn of Africa: the WFP HungerMap utilizes XGBoost regression-tree models[49] to nowcast[10] and forecast[50] food insecurity. In socioeconomic models, interpretability is crucial to generate trust from decision makers. This has been commonly achieved through computing SHAP values[51] after model training, for instance to discover drivers of displacement[52]. Now, causal machine learning is gaining traction with recent work leveraging causal inference methods[53] to predict displacement in Somalia[54], providing insights for humanitarian response and planning. Furthermore, symbolic regression is used to discover symbolic expressions directly from data, allowing for interpretatability[55-58]. Looking ahead, transformers, like the Perceiver-IO[59] and 4M[60] models, show promise in handling multiple modalities and long-range teleconnections. These models can combine diverse data, such as county-level agricultural data and high-

resolution satellite imagery, for applications like crop yield prediction[61]. Transformers also demonstrate success in leveraging circulation-driven teleconnections to forecast global wildfire risk[45], paving the way for improved EWS leveraging similar cross-boundary effects such as supply chains or streamflow.

The impact of AI on communication and the last mile challenge in EWSs remains uncertain. While it becomes challenging to envision how AI could assist vulnerable communities struggling with basic needs, there are specific communication challenges where AI may excel. Adjusting warnings to the local context, providing interactivity, and doing so in native languages are areas where AI, especially ChatBots, could be beneficial. These large language models (LLMs) are now good at in-context learning[62] meaning they can explain warnings in reasonable narratives and offer context-specific answers, as seen in flood disaster reporting[63]. Applying such models globally, especially in the global south with diverse languages, presents challenges [64]. Yet, recent work on multilingual language models[65,66] holds the potential to democratize EWS for historically under-served communities, facilitating easier communication of warnings in multiple languages at minimal cost. The next frontier lies in spoken language, where radio, identified by UN Global Pulse as a powerful tool for social listening[67], can be leveraged by speech models supporting over 1000+ languages[68] as additional data, paving the way for user-centric EWS.

AI is not a silver bullet to solve all problems – rather the usage of AI comes with its own challenges. In particular supervised ML, which seems most promising for EWS, suffers from problems with biases. Most prominently, supervised AI methods are particularly strong if the testing scenario is as close as possible to the training data — that is if there is no distribution shift between training and testing data[69]. However, in Earth-related datasets there is a sampling bias towards certain time periods and locations[70] and the climate extremes relevant for EWS are rare (though increasingly less so), hence distribution shifts are a common problem. Here, simulation[71], climate analogues[72], and space-for-time substitution[73] may be useful to obtain additional data samples and dampen distribution shifts. Furthermore, the data sets seldom contain all relevant variables (omitted variable bias) and often have data impurities, be it low sensor precision for physical variables or privacy and representativity issues with socioeconomic data. The former can be approached with multifidelity models involving data uncertainty[74], while the latter can be tackled through citizen science, that is involving volunteers in data collection, an approach that is successful in conservation ecology[75] and humanitarian mapping[76].

Data-related challenges can sometimes be even further amplified by inductive biases in DNNs. Inductive biases are modeling assumptions (e.g. locality or recurrence) that are needed to make deep learning work[77], but often cause unwanted side-effects. For instance most DNNs have a spectral bias — they prefer low-resolution features and omit high-frequency information[78]. This can lead to shortcut learning[79]: e.g. an image classification DNN may pick up non-causal features, such as using a green background to predict a cow, hence failing if a cow is in front of a blue background. Causal representation learning aims at building DNNs that are only using causal links in the data and thus capture the complex cause-and-effect relationships in the system[80]. While this area is still under active research, developing ML models and especially FMs for EWS should aim to observe causality. For instance, a causal model can evaluate the impact of interventions and thus understand the differences between the observed world and what might have occurred without humanitarian action. Additionally, causal models are interpretable by construction. For example, a causal model for wildfires could disentangle complex driving relations beyond spurious correlations, and could reveal an increased wildfire danger if, in addition to high heat, there is also prolonged water stress and specific socioeconomic conditions.

Moving towards FMs for EWS, an inherent property of generative models becomes a challenge: the generation of seemingly credible yet incorrect information termed hallucination[81]. In particular, EWSs that process natural language are vulnerable to hallucinate. Recent large language models[82,83] have tackled hallucination through scale and through Reinforcement Learning from Human Feedback (RLHF)[84]. The idea being larger data sets and expert knowledge can help improve correctness. Especially for a multimodal EWS FM, domain expertise ranging from meteorology, environmental sciences to sociology and politics is necessary to help refine the accuracy and relevance of generated information. Here, increasing the explainability of FMs by outputting intermediate results sequentially in a step-by-step manner is both necessary for validation and has been shown to decrease hallucination[85,86].

As it is, DNNs are frequently described as “black boxes”, due to their property of approximating an high-dimensional function that cannot be easily interpreted by humans. This may hinder their applicability in policy where trust is necessary. Especially at the beginning of the deployment of an AI model, trust can be earned if the reasoning behind model predictions can be explained[87]. For instance, if one were to study the effects of different humanitarian interventions modeled with what-if scenarios, which requires considering both direct effects and feedbacks, it is hard to imagine high trust in the predictions, if underlying reasons are not understood. So far, most approaches to explainable AI focus on post-hoc explanations[88] that are generated with a fully trained model. However, these models

are not trained for generating faithful attributions, so it is not ensured that the post-hoc explanations are interpretable[89]. Hence, other ways of directly encoding interpretable parts into the model, for instance through hybrid modeling, may be more suitable[90]. An interesting approach here is building up agent-based modeling, where stakeholders are represented as independent agents interacting with each other. Each agent could be an AI, with learned behavior, allowing to model socio-economic systems directly from raw data[91,92].

The early warning chain is a complex interconnected system with parts from different silos that build upon each other. Modern AI FMs may now offer the opportunity to overcome those silos and develop an integrated EWS. Such a system would be multi-hazard and multi-impact and span the whole warning chain including communication and decision making. In a first step, FMs that already exist (**Meteorological FMs** and **Geospatial FMs**), can be fine-tuned to improve individual pillars such as hazard or impact forecasting (Fig. 2). Second, they may be combined into **Impact FMs**, that can work with weather, geo-spatial and socioeconomic data to produce impact-forecasts in an integrated way. Finally, **Early Warning FMs** interface the impact model with natural language, photos and videos, hence bridging all modalities involved in the warning chain and thus achieving a fully integrated MHEWS.

Both Impact and Early Warning FMs need to work with multiple modalities. Initially, FMs were pre-trained on large and diverse datasets of single modalities, and subsequently fine-tuned for various tasks. They demonstrate remarkable “zero-shot” generalization, often accurately predicting outcomes for tasks they were not explicitly trained for. Further, the representations learned by these models enable them to adapt to new tasks using a minimal amount of additional data, in a process called fine-tuning. A wide variety of models used in natural language processing[82,83,93,94], computer vision[95-97] and speech processing[98] have provided empirical evidence for zero-shot generalization and fine-tuning across various data modalities. Recently, the pre-training approach has also seen success when combining multiple modalities[99-102], resulting in large multi-modal models (LMMs) that exhibit rich joint performance over text, image and speech. This development signifies a step towards more holistic, integrated AI systems that can process and interpret diverse data types. Instead of bridging images, text and speech, LMMs for EWS (Early Warning FMs) primarily process time-series data and maps of diverse physical and socioeconomic variables, various sensors, many resolutions, alongside natural language and photos for interactivity. Techniques like feature-wise linear modulation[103] and cross-attention[48,104] can be employed to fuse these modalities. Some of the geospatial FMs already use those methods to have first multi-modal capabilities[47].

However, such a model system for Early warning will have to obey important characteristics listed in Box 1. The design which best fits those criteria will need to be researched in the coming years. Crucially, models should be capable of simulating what-if scenarios, providing foresight into potential outcomes and enabling proactive decision-making (e.g. what happens if a floodgate gets opened, or if a village is evacuated). To this end, in addition to merely representing statistical dependencies, the models should learn causal representations that support prediction under interventions and that have been argued to be more robust with respect to ubiquitous distribution shifts[80]. Causal representations structure problems in terms of mechanisms underlying the data-generating process, and lend themselves well to building hybrid models or causal digital twins that combine components learned from data with other sources of knowledge, such as surrogate models or simulations[105]. This can be in conflict with the popular end-to-end learning, but it provides sequential "checkpoints" (e.g. prediction, then communication) which may additionally enhance model trust. Further considerations that may inform the design of those models is the level of explainability, transparency and recourse that they should provide. If decisions are taken by humans based on information provided by AI systems, it is crucial for a human to be able to understand the causes underlying a recommendation[87].

For the challenge of robustness, several training methodologies are promising. The "contrastive" learning technique is particularly prominent, aiming to harmonize data representations across varying modalities. It builds a latent space, where similar information is lumped closely together, irrespective of the underlying data modality, successfully integrating audio, images, text, and sensor data[99-102]. Alternatively, the latent space can also be build such that representations of complementary data inputs are predictive of each other, which enhances accuracy for uni-modal[106] and multi-modal[107] models alike. In parallel with advancements in large language models [82,94], reinforcement learning can be employed for further refinement. This process fine-tunes the pre-trained model through a rewards-based mechanism, focusing on the precision of its decisions and recommendations. In addition, this technique can be used to adapt the warning to the local cultural context and make it inclusive by thoughtful design under non-crises conditions, avoiding human biases readily occurring under stress[108]. Such a strategy is invaluable in crisis scenarios, leading to more effective and informed interventions.

In addition, to fully realize the potential of AI systems in EWS, they should be context-specific and designed with user-friendly interfaces that can be readily deployed by front-line humanitarian workers with limited technical expertise. Ideally, such systems should be able

to function as a contributing participant in crisis meetings, providing real-time data analysis, predictive modeling, and actionable insights to inform decision-making. Hence, the resulting system in its entirety would comprise one or many FMs doing the heavy lifting, complemented with domain specific models and expertise for robustness, interpretability and intervention analysis and amended with text and image processing for user-centric communication, interactivity and active learning feedbacks (Fig. 3).

However, one key limiting factor is the collection and management of training data for these models. Unlike for text or images, there are no large, harmonized datasets for multi-modal EWS. Instead, many different sources need to be combined. While some, such as ESA's Sentinel 2 satellite imagery[109] or ECMWFs ERA5 meteorological reanalysis[19] are large publicly available datasets in analysis-ready formats, others are not. An Impact FM will require gridded and tabular socio-economic data such as crop yield or hospitalizations, which are seldom standardized across administrative boundaries and also of much smaller quantity (Gigabytes instead of Petabytes). For Early Warning FMs, alongside standard text datasets, more specialized resources such as humanitarian reports or press articles, which are scattered across the internet and not always public, need to be collected. Hence, the multi-modal systems for EWS will largely rely on pre-training with those large-scale datasets that do exist, and then careful addition of sparser data sources, e.g. using positional meta data or building upon natural language as a mediating modality.

Validation and verification of EWSs for climate hazards pose important challenges. On the one hand, FMs present unique challenges in validation due to their unprecedented versatility. Traditional AI models in geoscience are often designed for specific tasks, such as predicting rainfall patterns or detecting signs of drought from satellite imagery. These models are validated against these specific use cases. However, FMs are capable of performing a wide range of tasks, including those that might be specified by an end-user for the first time, like predicting the impact of an unforeseen climatic event. This broader scope makes it inherently more challenging to anticipate all potential failure modes. Developers and regulators will need to clearly communicate the tested use cases for which FMs warning systems are validated and caution users against 'off-label usage' that ventures into new, untested territories. The broad capabilities of such Early Warning FMs require regulatory foresight, necessitating adaptations in institutional and governmental policies, and may also influence insurance and liability frameworks. This complexity may require assessments by multidisciplinary teams including climatologists, meteorologists, environmental scientists, and other specialists, making the fact-checking process more challenging both during validation and post-deployment. On the other hand, in order to facilitate the verification of Early Warning FMs

outputs, developers should ideally incorporate explainability techniques. For instance, outputs could include references or links to underlying data sources or scientific literature that support the model's predictions. This would allow experts to more efficiently verify the accuracy and reliability of Early Warning FMs predictions. Furthermore, it is crucial for Early Warning FMs to accurately express uncertainties in their predictions to prevent overconfident and potentially misleading statements.

Last but not least, current early warning systems, which are based on impacts caused by concrete weather conditions in the next hours to weeks, should be complemented by decadal time-scale early warning systems. Developing a decadal time-scale EWS for climate and weather risks is essential due to the increasing variability and extremity of weather patterns caused by climate change. Decadal EWS should guide effective adaptation measure, more targeted than what can be inferred from general climate change metric and a general precautionary principle. This involves identifying vulnerable regions and sectors, planning infrastructure developments, and formulating policies that are resilient to long-term climatic changes. Effective communication strategies are needed to convey long-term risks and adaptations to governments, businesses, and communities, ensuring preparedness. Of course, reliable forecasts are a prerequisite, too. Hence, generally similar challenges as the ones mentioned above for short-term EWS need to be addressed, yet with an important addition: For this important challenge probabilistic forecasts are highly relevant. These forecasts present a range of possible outcomes with associated probabilities, offering a more nuanced understanding of long-term risks. In addition, there has been a trade-off between the spatial resolution of predictions and the time scale over which they are made – longer time scales typically meant broader, less detailed spatial predictions (Fig. 4). However, probabilistic AI may disrupt this norm, and generate high-resolution forecasts even for extended time scales. This can be possible, because AI can effectively model and account for the even aleatoric uncertainties inherent in long-term forecasts. This approach provides a more detailed and nuanced understanding of potential future scenarios, even at a granular spatial level. For instance, it can provide localized climate-risk assessments for specific regions or cities far into the future, something that was traditionally challenging due to the broad-brush approach required for long-term forecasts. In essence, probabilistic AI breaks the conventional link between spatial and temporal scales in forecasting, enabling more precise and detailed long-term predictions. This advancement is crucial for effective risk assessment and planning in the context of climate change and weather variability.

Supplementary references:

1 Mohr, S. *et al.* A multi-disciplinary analysis of the exceptional flood event of July 2021 in central Europe. Part 1: Event description and analysis. *NHESSD 2, 5079–5111, 2014 Seasonal forecasting of fire over Kalimantan, Indonesia A. C. Spessa et al. Title Page Abstract Introduction Conclusions References Tables Figures J I J I Back Close Full Sc* **2022**, 1-44 (2022).

2 Ludwig, P. *et al.* A multi-disciplinary analysis of the exceptional flood event of July 2021 in central Europe–Part 2: Historical context and relation to climate change. *Natural Hazards and Earth System Sciences* **23**, 1287-1311 (2023).

3 Koks, E., Van Ginkel, K., Van Marle, M. & Lemnitzer, A. Brief communication: Critical infrastructure impacts of the 2021 mid-July western European flood event. *NHESSD 2, 5079–5111, 2014 Seasonal forecasting of fire over Kalimantan, Indonesia A. C. Spessa et al. Title Page Abstract Introduction Conclusions References Tables Figures J I J I Back Close Full Sc* **2021**, 1-11 (2021).

4 Mohr, S. *et al.* A multi-disciplinary analysis of the exceptional flood event of July 2021 in central Europe – Part 1: Event description and analysis. *Nat. Hazards Earth Syst. Sci.* **23**, 525-551 (2023). https://doi.org/10.5194/nhess-23-525-2023

5 Cornwall, W. Europe's deadly floods leave scientists stunned. *Science* **373**, 372-373 (2021). https://doi.org/doi:10.1126/science.373.6553.372

6 Economist, T. Canada's wildfires have burnt an area 16 times larger than normal. https://www.economist.com/the-americas/2023/06/15/canadas-wildfires-have-burnt-an-area-16-times-larger-than-normal. (2023).

7 Kelly, J. Record breaking PM2.5 pollution levels in NYC in early June 2023 regular occurrence in over 350 cities worldwide. https://energyandcleanair.org/record-breaking-pm2-5-pollution-levels-in-nyc-in-early-june-2023-regular-occurrence-in-over-350-cities-worldwide/#:~:text=Record%20breaking%20PM2.-,5%20pollution%20levels%20in%20NYC%20in%20early%20June%202023,in%20over%20350%.). (2023).

8 https://www.nyc.gov/assets/doh/downloads/pdf/eode/eode-air-quality-impact.pdf, N. Y. C. D. o. H. a. M. H. Air Pollution and the Health of New Yorkers. 40.

9 CEMS. *FAQ on EFAS and the recent flood events*, <https://www.efas.eu/en/news/faq-efas-and-recent-flood-events> (2021).

10 Martini, G. *et al.* Machine learning can guide food security efforts when primary data are not available. *Nature Food* **3**, 716-728 (2022).

11 Kochkov, D. *et al.* Machine learning–accelerated computational fluid dynamics. *Proceedings of the National Academy of Sciences* **118**, e2101784118 (2021).

12 Robin, C. *et al.* preprint - Learning to forecast vegetation greenness at fine resolution over Africa with ConvLSTMs. (2022).

13 Balashankar, A., Subramanian, L. & Fraiberger, S. P. Predicting food crises using news streams. *Science Advances* **9**, eabm3449 (2023).

14 Kochkov, D. *et al.* Neural General Circulation Models. (2023). https://doi.org/10.48550/arXiv.2311.07222

15 Lam, R. *et al.* Learning skillful medium-range global weather forecasting. *Science* **0**, eadi2336 (2023). https://doi.org/10.1126/science.adi2336

16 Bi, K. *et al.* Accurate medium-range global weather forecasting with 3D neural networks. *Nature* **619**, 533-538 (2023). https://doi.org/10.1038/s41586-023-06185-3

17 Pathak, J. *et al.* FourCastNet: A Global Data-driven High-resolution Weather Model using Adaptive Fourier Neural Operators. (2022). https://doi.org/10.48550/arXiv.2202.11214

18 Nguyen, T., Brandstetter, J., Kapoor, A., Gupta, J. K. & Grover, A. ClimaX: A Foundation Model for Weather and Climate. *1st workshop on Synergy of Scientific and Machine Learning Modeling, SynS & ML ICML* (2023).

19 Hersbach, H. *et al.* The ERA5 global reanalysis. *Quarterly Journal of the Royal Meteorological Society* **146**, 1999-2049 (2020). https://doi.org/https://doi.org/10.1002/qj.3803

20 Ravuri, S. *et al.* Skilful precipitation nowcasting using deep generative models of radar. *Nature* **597**, 672-677 (2021). https://doi.org/10.1038/s41586-021-03854-z

21 Gao, Z. *et al.* (2022).

22 Espeholt, L. *et al.* Deep learning for twelve hour precipitation forecasts. *Nature Communications* **13**, 5145 (2022). https://doi.org/10.1038/s41467-022-32483-x

23 Asperti, A. *et al.* Precipitation nowcasting with generative diffusion models. (2023). https://doi.org/10.48550/arXiv.2308.06733

24 Shi, X. *et al.* Convolutional LSTM network: A machine learning approach for precipitation nowcasting. *Advances in neural information processing systems* **28** (2015).

25 Zhang, Y. *et al.* Skilful nowcasting of extreme precipitation with NowcastNet. *Nature* **619**, 526-532 (2023). https://doi.org/10.1038/s41586-023-06184-4

26 Lam, R. *et al.* Learning skillful medium-range global weather forecasting. *Science*, eadi2336 (2023).

27 Thuemmel, J. *et al.* Inductive biases in deep learning models for weather prediction. (2023). https://doi.org/10.48550/arXiv.2304.04664

28 Dosovitskiy, A. *et al. An Image is Worth 16x16 Words: Transformers for Image Recognition at Scale*, <https://openreview.net/forum?id=YicbFdNTTy> (2023).

29 Keisler, R. Forecasting Global Weather with Graph Neural Networks. (2022). https://doi.org/10.48550/arXiv.2202.07575

30 Ham, Y.-G., Kim, J.-H. & Luo, J.-J. Deep learning for multi-year ENSO forecasts. *Nature* **573**, 568-572 (2019). https://doi.org/10.1038/s41586-019-1559-7

31 Petersik, P. J. & Dijkstra, H. A. in *Geophys Res Lett* Vol. 47 8 (2020).

32 Dijkstra, H., Petersik, P., Hernández-García, E. & López, C. The Application of Machine Learning Techniques to Improve El Niño Prediction Skill. *Frontiers in Physics*, 13 (2019). https://doi.org/10.3389/fphy.2019.00153

33 Cachay, S. R. *et al.* The World as a Graph: Improving El Ni\~no Forecasts with Graph Neural Networks. (2021). https://doi.org/10.48550/arXiv.2104.05089

34 Schlör, J., Strnad, F., Capotondi, A. & Goswami, B. A multi-modal representation of El Ni\~no Southern Oscillation Diversity. (2023). https://doi.org/10.48550/arXiv.2307.11552

35 Funk, C. *et al.* Frequent but Predictable Droughts in East Africa Driven by a Walker Circulation Intensification. *Earth's Future* **11**, e2022EF003454 (2023). https://doi.org/https://doi.org/10.1029/2022EF003454

36 Kratzert, F. *et al.* Towards learning universal, regional, and local hydrological behaviors via machine learning applied to large-sample datasets. *Hydrol. Earth Syst. Sci.* **23**, 5089-5110 (2019). https://doi.org/10.5194/hess-23-5089-2019

37 Nearing, G. *et al.* AI Increases Global Access to Reliable Flood Forecasts. *arXiv preprint arXiv:2307.16104* (2023).
38 Nevo, S. *et al.* Flood forecasting with machine learning models in an operational framework. *Hydrol. Earth Syst. Sci.* **26**, 4013-4032 (2022). https://doi.org/10.5194/hess-26-4013-2022
39 Barrett, A. B. *et al.* Forecasting vegetation condition for drought early warning systems in pastoral communities in Kenya. *Remote Sensing of Environment* **248**, 111886 (2020). https://doi.org/https://doi.org/10.1016/j.rse.2020.111886
40 Benson, V. *et al.* Forecasting localized weather impacts on vegetation as seen from space with meteo-guided video prediction. (2023).
41 Robin, C. *et al.* in *Artificial Intelligence for Humanitarian Assistance and Disaster Response workshop at NeurIPS* (2022).
42 Requena-Mesa, C., Benson, V., Denzler, J., Runge, J. & Reichstein, M. EarthNet2021: A novel large-scale dataset and challenge for forecasting localized climate impacts. Accepted at Tackling Climate Change with Machine Learning workshop at NeurIPS. *https://arxiv.org/abs/2012.06246v1* (2020).
43 Smith, M. J., Fleming, L. & Geach, J. E. EarthPT: a foundation model for Earth Observation. *arXiv preprint arXiv:2309.07207* (2023).
44 Zafeiropoulos, K. *Greece's map for predicting wildfires is anachronistic and inadequate*, <https://miir.gr/en/greece-s-map-for-predicting-wildfires-is-anachronistic-and-inadequate/> (2023).
45 Prapas, I. *et al.* TeleViT: Teleconnection-driven Transformers Improve Subseasonal to Seasonal Wildfire Forecasting. 6 (2023).
46 Jakubik, J. *et al.* Foundation Models for Generalist Geospatial Artificial Intelligence. *arXiv preprint arXiv:2310.18660* (2023).
47 Tseng, G., Zvonkov, I., Purohit, M., Rolnick, D. & Kerner, H. Lightweight, Pre-trained Transformers for Remote Sensing Timeseries. *arXiv preprint arXiv:2304.14065* (2023).
48 Rombach, R., Blattmann, A., Lorenz, D., Esser, P. & Ommer, B. in *Proceedings of the IEEE/CVF Conference on Computer Vision and Pattern Recognition.* 10684-10695.
49 Chen, T. & Guestrin, C. in *Proceedings of the 22nd ACM SIGKDD International Conference on Knowledge Discovery and Data Mining* 785–794 (Association for Computing Machinery, San Francisco, California, USA, 2016).
50 Foini, P., Tizzoni, M., Martini, G., Paolotti, D. & Omodei, E. On the forecastability of food insecurity. *Scientific Reports* **13**, 2793 (2023).
51 Lundberg, S. M. & Lee, S.-I. (Curran Associates, Inc.).
52 Ronco, M. *et al.* Exploring interactions between socioeconomic context and natural hazards on human population displacement. *Nat Commun* **14**, 8004 (2023). https://doi.org/10.1038/s41467-023-43809-8
53 Runge, J. *et al.* Inferring causation from time series in Earth system sciences. *Nature Communications* **10**, 2553 (2019). https://doi.org/10.1038/s41467-019-10105-3
54 Tárraga Habas, J. M., Sevillano Marco, E. & Miranda, M. T. The state-of-the-art on Drought Displacement Modelling. (iDMC internal displacement monitoring centre, 2022).
55 Brunton, S. L., Proctor, J. L. & Kutz, J. N. Discovering governing equations from data by sparse identification of nonlinear dynamical systems. *Proceedings of the National Academy of Sciences* **113**, 3932-3937 (2016). https://doi.org/10.1073/pnas.1517384113
56 Cranmer, M. Interpretable Machine Learning for Science with PySR and SymbolicRegression.jl. (2023). https://doi.org/10.48550/arXiv.2305.01582
57 Cranmer, M. *et al.* 17429–17442 (Curran Associates, Inc.).

58 Camps-Valls, G. *et al.* Discovering causal relations and equations from data. *Physics Reports* **1044**, 1-68 (2023). https://doi.org/10.1016/j.physrep.2023.10.005
59 Jaegle, A. *et al.* in *ICLR 2022* (2022).
60 Mizrahi, D. *et al.* in *Neural Information Processing Systems.* (arXiv).
61 Lin, F. *et al.* in *Proceedings of the IEEE/CVF International Conference on Computer Vision (ICCV)* pp. 5774-5784 (2023).
62 Brown, T. *et al.* in *Advances in Neural Information Processing Systems 33 (NeurIPS 2020)* (2020).
63 Colverd, G., Darm, P., Silverberg, L. & Kasmanoff, N. in *6th Workshop on Artificial Intelligence for Humanitarian Assistance and Disaster Response (NeurIPS 2023).* (arXiv).
64 Naudé, W. & Vinuesa, R. Data deprivations, data gaps and digital divides: Lessons from the COVID-19 pandemic. *Big Data & Society* **8**, 20539517211025545 (2021). https://doi.org/10.1177/20539517211025545
65 Ogueji, K., Zhu, Y. & Lin, J. in *MRL 2021.* (eds Duygu Ataman *et al.*) 116–126 (Association for Computational Linguistics).
66 Wei, X. *et al.* PolyLM: An Open Source Polyglot Large Language Model. (2023). https://doi.org/10.48550/arXiv.2307.06018
67 Luccioni, A. S., Pham, K. H., Lam, C. S. N., Aylett-Bullock, J. & Luengo-Oroz, M. (eds Michael Kamp *et al.*) 259-266 (Springer International Publishing).
68 Pratap, V. *et al.* Scaling Speech Technology to 1,000+ Languages. (2023). https://doi.org/10.48550/arXiv.2305.13516
69 Schneider, S. *et al.* Improving robustness against common corruptions by covariate shift adaptation. *Advances in neural information processing systems* **33**, 11539-11551 (2020). https://doi.org/10.48550/arXiv.2006.16971
70 Meyer, H. & Pebesma, E. Machine learning-based global maps of ecological variables and the challenge of assessing them. *Nature Communications* **13**, 1-4 (2022).
71 Kekić, A. *et al.* Evaluating vaccine allocation strategies using simulation-assisted causal modeling. *Patterns* **4**, 100739 (2023). https://doi.org/https://doi.org/10.1016/j.patter.2023.100739
72 Hallegatte, S., Hourcade, J.-C. & Ambrosi, P. Using climate analogues for assessing climate change economic impacts in urban areas. *Climatic Change* **82**, 47-60 (2007). https://doi.org/10.1007/s10584-006-9161-z
73 Pickett, S. T. A. in *Long-Term Studies in Ecology: Approaches and Alternatives* (ed Gene E. Likens) 110-135 (Springer New York, 1989).
74 Morimoto, M., Fukami, K., Maulik, R., Vinuesa, R. & Fukagata, K. Assessments of epistemic uncertainty using Gaussian stochastic weight averaging for fluid-flow regression. *Physica D: Nonlinear Phenomena* **440**, 133454 (2022). https://doi.org/10.1016/j.physd.2022.133454
75 Silvertown, J. A new dawn for citizen science. *Trends in Ecology & Evolution* **24**, 467-471 (2009). https://doi.org/10.1016/j.tree.2009.03.017
76 Herfort, B., Lautenbach, S., Porto de Albuquerque, J., Anderson, J. & Zipf, A. The evolution of humanitarian mapping within the OpenStreetMap community. *Scientific Reports* **11**, 3037 (2021). https://doi.org/10.1038/s41598-021-82404-z
77 Goyal, A. & Bengio, Y. Inductive biases for deep learning of higher-level cognition. *Proceedings of the Royal Society A* **478**, 20210068 (2022).
78 Ackaah-Gyasi, K. N., Valdez, S., Gao, Y. & Zhang, L. Exploring Spectral Bias in Time Series Long Sequence Forecasting. 6 (2023).
79 Geirhos, R. *et al.* Shortcut learning in deep neural networks. *Nature Machine Intelligence* **2**, 665-673 (2020).
80 Schölkopf, B. *et al.* Toward Causal Representation Learning. *Proceedings of the IEEE* **109**, 612-634 (2021). https://doi.org/10.1109/JPROC.2021.3058954

81 Ji, Z. *et al.* Survey of hallucination in natural language generation. *ACM Computing Surveys* **55**, 1-38 (2023).
82 OpenAI *et al.* GPT-4 Technical Report. (2023). https://doi.org/10.48550/arXiv.2303.08774
83 Chowdhery, A. *et al.* PaLM: Scaling Language Modeling with Pathways. *Journal of Machine Learning Research* **24**, 1-113 (2023).
84 Ouyang, L. *et al.* Training language models to follow instructions with human feedback. *Advances in Neural Information Processing Systems* **35**, 27730-27744 (2022).
85 Dhuliawala, S. *et al.* Chain-of-verification reduces hallucination in large language models. *arXiv preprint arXiv:2309.11495* (2023).
86 Pan, L. *et al.* Automatically correcting large language models: Surveying the landscape of diverse self-correction strategies. *arXiv preprint arXiv:2308.03188* (2023).
87 Vinuesa, R. & Sirmacek, B. Interpretable deep-learning models to help achieve the Sustainable Development Goals. *Nature Machine Intelligence* **3**, 926-926 (2021).
88 Samek, W., Montavon, G., Lapuschkin, S., Anders, C. J. & Müller, K.-R. Explaining deep neural networks and beyond: A review of methods and applications. *Proceedings of the IEEE* **109**, 247-278 (2021).
89 Ronco, M. & Camps-Valls, G. Role of locality, fidelity and symmetry regularization in learning explainable representations. *Neurocomputing* **562**, 126884 (2023). https://doi.org/https://doi.org/10.1016/j.neucom.2023.126884
90 Reichstein, M. *et al.* Deep learning and process understanding for data-driven Earth system science. *Nature* **566**, 195-204 (2019). https://doi.org/10.1038/s41586-019-0912-1
91 Zheng, S., Trott, A., Srinivasa, S., Parkes, D. C. & Socher, R. The AI Economist: Taxation policy design via two-level deep multiagent reinforcement learning. *Science advances* **8**, eabk2607 (2022).
92 Park, J. S. *et al.* in *Proceedings of the 36th Annual ACM Symposium on User Interface Software and Technology.* 1-22.
93 Radford, A., Narasimhan, K., Salimans, T. & Sutskever, I. Improving Language Understanding by Generative Pre-Training. (2018).
94 Touvron, H. *et al.* Llama 2: Open Foundation and Fine-Tuned Chat Models. (2023). https://doi.org/10.48550/arXiv.2307.09288
95 Dosovitskiy, A. *et al.* in *International Conference on Learning Representations.*
96 Kirillov, A. *et al.* in *Proceedings of the IEEE/CVF International Conference on Computer Vision.* 4015-4026.
97 Oquab, M. *et al.* DINOv2: Learning Robust Visual Features without Supervision. *Transactions on Machine Learning Research* (2023). https://doi.org/10.48550/arXiv.2304.07193
98 Radford, A. *et al.* in *International Conference on Machine Learning.* 28492-28518 (PMLR).
99 Radford, A. *et al.* in *International Conference on Machine Learning.* 8748-8763 (PMLR).
100 Girdhar, R. *et al.* in *Proceedings of the IEEE/CVF Conference on Computer Vision and Pattern Recognition.* 15180-15190.
101 Alayrac, J.-B. *et al.* Flamingo: a Visual Language Model for Few-Shot Learning. *Advances in Neural Information Processing Systems* **35**, 23716-23736 (2022).
102 Liu, H., Li, C., Wu, Q. & Lee, Y. J. in *Neural Information Processing Systems.* (arXiv).
103 Perez, E., Strub, F., Vries, H. d., Dumoulin, V. & Courville, A. FiLM: Visual Reasoning with a General Conditioning Layer. *Proceedings of the AAAI Conference on Artificial Intelligence* **32** (2018). https://doi.org/10.1609/aaai.v32i1.11671
104 Zhang, Y. & Yan, J. in *The Eleventh International Conference on Learning Representations.*

105 Kekić, A. *et al.* Evaluating vaccine allocation strategies using simulation-assisted causal modeling. *Patterns* **4** (2023). https://doi.org/10.1016/j.patter.2023.100739
106 Oord, A. v. d., Li, Y. & Vinyals, O. Representation Learning with Contrastive Predictive Coding. (2019). https://doi.org/10.48550/arXiv.1807.03748
107 Assran, M. *et al.* in *2023 IEEE/CVF Conference on Computer Vision and Pattern Recognition (CVPR).* 15619-15629.
108 Yu, R. Stress potentiates decision biases: A stress induced deliberation-to-intuition (SIDI) model. *Neurobiology of stress* **3**, 83-95 (2016).
109 Louis, J. *et al.* in *ESA Living Planet Symposium 2016.* (ed L. Ouwehand) 1-8 (Spacebooks Online).
